# A Conceptual Data Model of Datum Systems

**DOI:** 10.6028/jres.104.024

**Published:** 1999-08-01

**Authors:** Michael R. McCaleb

**Affiliations:** National Institute of Standards and Technology, Gaithersburg, MD 20899-0001

**Keywords:** data model, datum, datum feature, datum system, datum target

## Abstract

A new conceptual data model that addresses the geometric dimensioning and tolerancing concepts of datum systems, datums, datum features, datum targets, and the relationships among these concepts, is presented. Additionally, a portion of a related data model, Part 47 of STEP (ISO 10303-47), is reviewed and a comparison is made between it and the new conceptual data model.

## 1. Introduction

Traditionally, geometric dimensioning and tolerancing (GD&T) requirements have been exchanged with technical drawings. However, with the advent of computer-aided design, manufacturing, and inspection equipment, the ability to exchange these requirements in a computer-sensible manner has become increasingly more desirable. As “a data model is an effective technique to define the shareable semantics that are essential to the success of data communication in an integrated environment” [1], a conceptual data model has been developed that defines a portion of the semantics necessary for the electronic exchange of GD&T data among the design, manufacturing, and inspection divisions of an enterprise. The portion of the semantics that this data model defines encompasses the concepts of datum systems, datums, datum features, and datum targets. This paper presents this data model, which will be referred to throughout the remainder of the paper as the DSCDM (Datum System Conceptual Data Model). Additionally, a portion of the data model presented in STEP Part 47 [2] is reviewed and a comparison is made between it and the DSCDM. The model presented in STEP Part 47 will be referred to as “the Part 47 model” throughout the remainder of this paper.

NOTE—Though the scope of the DSCDM is limited to the concepts mentioned above, the aim is to provide a foundation upon which more comprehensive GD&T data models may be based.
NOTE—The following conventions are employed throughout the course of this paper. To distinguish between EXPRESS entities and the objects they represent, entity names are printed in bold type and the objects they represent are printed in non-bold type. Furthermore, entity names start with a leading uppercase letter (e.g., **Datum** is an entity name and datum refers to the object). Attribute names are printed in italic type (e.g., *established_datum*). Additionally, permissible values from enumerated data types are printed in all uppercase letters (e.g., MAXIMUM_MATERIAL_PRINCIPLE).
NOTE—The data models in this paper are presented in EXPRESS-G notation. EXPRESS-G is a graphical notation that supports a subset of the EXPRESS data modelling language. Both EXPRESS and EXPRESS-G are defined in ISO 10303-11 [3]. An overview of the EXPRESS-G notation is presented in Appendix A of this paper as an aid to those who are unfamiliar with EXPRESS-G.

## 2. Requirements

“The first step in data modeling is to define the data requirements” [1]. In regard to the DSCDM, the requirements came from existing GD&T drawing-based standards (e.g., ASME Y14.5M [4], ISO 1101 [5], and ISO 5459 [6]). The reason that these existing GD&T standards are used to define the requirements of the DSCDM is due in part to the fact that the DSCDM is based on parts of a larger GD&T model that the author developed for STEP AP 210, *Electronic assembly, interconnect and packaging design* [7]. With the increased geometric complexity of printed circuit boards, printed circuit assemblies, and electronic components, it was deemed by members of the STEP AP 210 development team that the concepts presented in these GD&T drawing-based standards that are typically considered applicable to mechanical products are also applicable to the electronic products to which STEP AP 210 pertains. Consequently, these drawing-based standards define the main requirements of the GD&T model and subsequently of the DSCDM. Consequently, most of the definitions in Sec. 3 of this paper, which define the concepts that form the basis for the requirements of the DSCDM, are from these standards and associated reference books. Furthermore, most of the diagrams and examples presented in this paper are from these same sources. These diagrams and examples not only aid in explaining the DSCDM, but also provide a set of test cases by which the validity of both the DSCDM and the Part 47 model may be judged.

## 3. Geometric Dimensioning and Tolerancing Definitions

Most of the following definitions are from existing drawing-based GD&T standards and associated reference books. These definitions are important, because they explain some of the concepts that are at the foundation of these GD&T standards, and consequently form the basis for the requirements of the DSCDM.

Datum: “A theoretically exact point, axis, or plane derived from the true geometric counterpart of a specified datum feature. A datum is the origin from which the location or geometric characteristics of features of a part are established” [4].

Datum Feature: “An actual feature of a part that is used to establish a datum” [4].

Datum Feature Symbol: “The symbolic means of indicating a datum feature consists of a capital letter enclosed in a square frame and a leader line extending from the frame to the concerned feature, terminating with a triangle” [4].

Datum System: “A group of two or more separate datums used as a combined reference for a toleranced feature” [6].

Datum Reference Frame: A framework that consists of three mutually perpendicular datum planes, three datum axes (located at the intersection of each pair of datum planes), and a datum point (that is located at the intersection of the three datum planes).

Datum Target: “A specified point, line, or area on a part used to establish a datum” [4].

Datum Target Frame: “The datum targets are indicated by a circular frame divided in two compartments by a horizontal line. The lower compartment is reserved for a letter and a digit. The letter represents the datum feature and the digit the datum target number. The upper compartment is reserved for additional information, such as dimensions of the target area. If there is not sufficient space within the compartment, the information may be placed outside and connected to the appropriate compartment by a leader line” [6].

Feature: “The general term applied to a physical portion of a part, such as a surface, pin, tab, hole, or slot” [4].

Feature Control Frame: “The feature control frame is a rectangular box containing the geometric characteristic symbol and the form, orientation, profile, runout, or location tolerance. If necessary, datum references and modifiers applicable to the feature or the datums are also contained in the box, e.g.” 
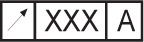
 [8].

Feature of Size: “One cylindrical or spherical surface, or a set of two opposed elements or opposed parallel surfaces, associated with a size dimension” [4].

Least Material Condition (LMC): “The condition in which a feature of size contains the least amount of material within the stated limits of size—for example, maximum hole diameter, minimum shaft diameter” [4].

Least Material Requirement: “The *least material requirement* permits an increase in the stated geometrical tolerance when the concerned feature departs from its least material condition (LMC)” [9].

Maximum Material Condition (MMC): “The condition in which a feature of size contains the maximum amount of material within the stated limits of size—for example, minimum hole diameter, maximum shaft diameter” [4].

Maximum Material Principle: “The *maximum material principle* is a tolerancing principle which requires that the virtual condition for the toleranced feature(s) and, if indicated, the maximum material condition of perfect form for datum feature(s), shall not be violated” [10].

Regardless of Feature Size (RFS): “The term used to indicate that a geometric tolerance or datum reference applies at any increment of size of the feature within its size tolerance” [4].

## 4. The Datum System Conceptual Data Model

STEP integrated generic resources are a series of STEP parts that define resource constructs that are context-independent. The underlying structure of the DSCDM is based on four entities from the STEP integrated generic resources. These entities are **Shape_aspect**, **Shape_aspect_relationship**, **Property_definition**, and **Property_definition_relationship**. A review of these entities is presented in Appendix B of this paper.

**Fig. 1:**
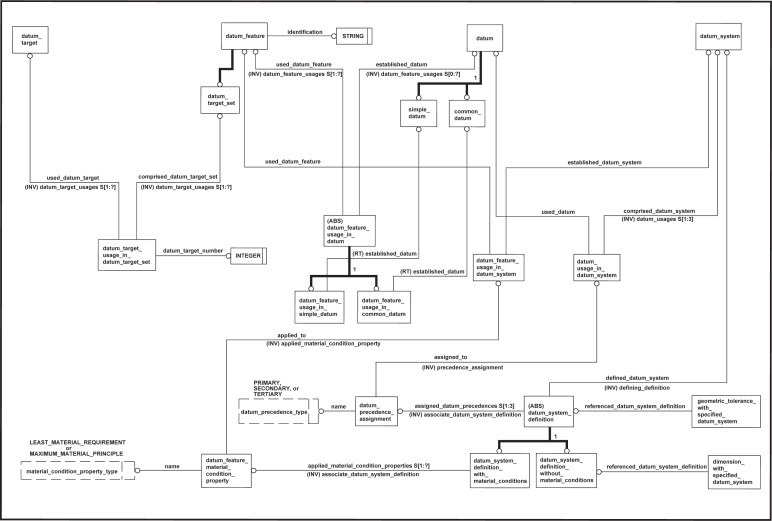
EXPRESS-G diagram of the DSCDM.

The DSCDM is presented in the EXPRESS-G diagram shown in Fig. 1. The entities are organized on the page such that the entities based on the **Shape_aspect** entity of STEP Part 41 [11] are at the top of the page. Immediately below the **Shape_aspect** based entities are the entities based on the **Shape_aspect_relationship** entity of STEP Part 41. At the bottom of the page are the entities based on the **Property_definition** entity of STEP Part 41. Note that the DSCDM does not actually contain entities based on the **Property_definition_relationship** entity of STEP Part 45 [12]. Instead, in the interest of simplicity, **Property_definition** based entities are related with attributes that have been included in the **Property_definition** based entities. For example, instead of specifying a **Property_definition_relationship** based entity in the DSCDM to relate the **Datum_system_definition** entity with the **Datum_precedence_assignment** entity, the relationship between these two entities is established by the *assigned_datum_precedences* attribute of the **Datum_system_definition** entity.

NOTE—While no **Property_definition_relationship** based entities exist in the DSCDM, they exist in spirit wherever two **Property_definition** based entities are related.

The definitions of the entities presented in Fig. 1 are defined below. These definitions are presented in the order they appear on the page. That is, the **Shape_aspect** based entities are first, followed by the **Shape_aspect_relationship** based entities, and finally, the **Property_definition** based entities.

### 4.1 Datum_system

A **Datum_system** corresponds to a datum system (see Sec. 3 of this paper) that is comprised of one to three datums.

NOTE—The **Datum_system** entity is based on the **Shape_aspect** entity of STEP Part 41 [11].
NOTE—The definition of datum system as defined in ISO 5459-1981 is given in Sec. 3 of this paper. However, for the purpose of this model, the definition of datum system has been extended so that a datum system may be comprised of a single datum.

#### Inverse attribute definitions

*datum_usages:* The *datum_usages* attribute specifies a set of one to three **Datum_usage_in_datum_system**s. Each of the **Datum_usage_in_datum_system**s in this set corresponds to the usage of a datum in the datum system.

*defining_definition:* The *defining_definition* attribute specifies the **Datum_system_definition** that specifies the characteristics of the corresponding datum system (e.g., the order in which each datum is established within the datum system).

**Fig. 2:**
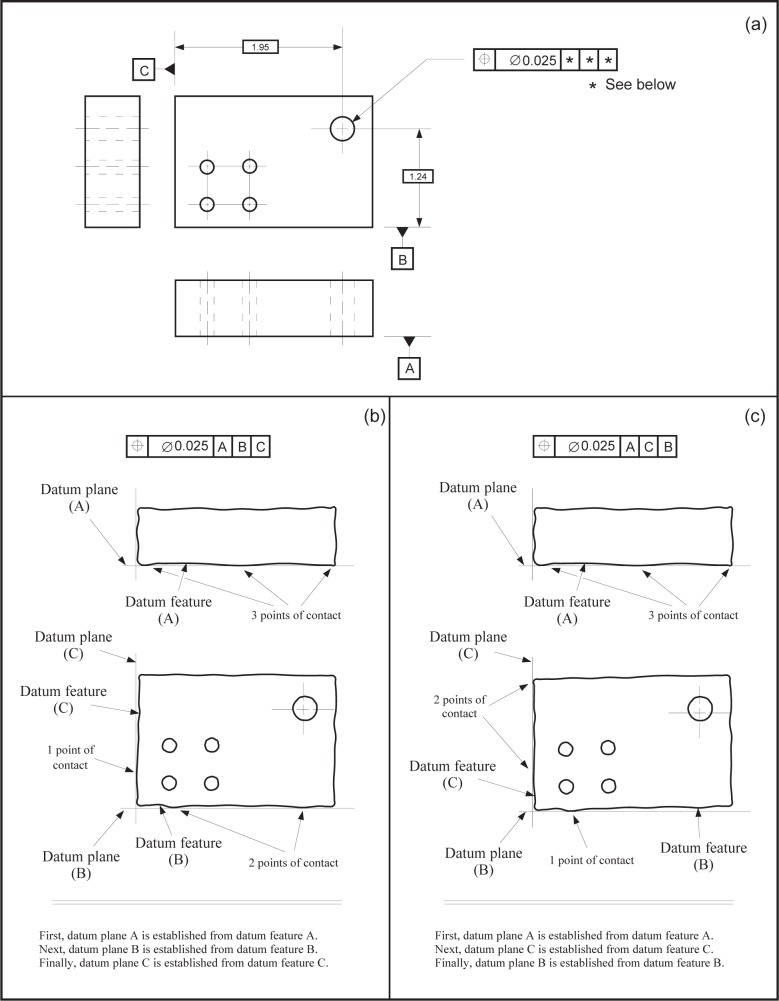
Effects of datum precedence on a datum system.

NOTE—On technical drawings, the characteristics of a datum system are typically specified in a feature control frame.
EXAMPLE—Both Figs. 2 (b) and (c) contain a feature control frame, each of which specifies a datum system that consists of three datums (datums A, B, and C). However, these two datum systems are different, as the order that the datums are established within each datum system differs (i.e., they have a different datum precedence). Figure 2 illustrates the effect that datum precedence has on a datum system.

#### Constraints

WR1: Of the **Datum_feature**s specified as the *used_datum_feature* by the **Datum_feature_usage_in_datum**s that are specified as the *datum_feature_usages* by the **Datum**s that are specified as the *used_datum* by the **Datum_usage_in_datum_system**s that are specified as the *datum_usages* of the **Datum_system**, no **Datum_feature** may be specified more than once.

NOTE—WR1 corresponds to the assertion that each datum feature shall not be used more than once in establishing any one datum system.

WR2: Of the **Datum_target**s specified as the *used_datum_target* by the **Datum_target_usage_in_datum_target_set**s specified as the *datum_target_usages* by the **Datum_target_set**s specified as the *used_datum_feature* by the **Datum_feature_usage_in_datum**s that are specified as the *datum_feature_usages* by the **Datums** that are specified as the *used_datum* by the **Datum_usage_in_datum_system**s that are specified as the *datum_usages* of the **Datum_system**, no **Datum_target** may be specified more than once.

NOTE—WR2 corresponds to the assertion that each datum target shall not be used more than once in establishing any one datum system.

### 4.2 Datum

A **Datum** corresponds to a datum (see Sec. 3 of this paper). A **Datum** may be either a **Simple_datum** or a **Common_datum**.

NOTE—The **Datum** entity is based on the **Shape_aspect** entity of STEP Part 41 [11].

#### Inverse attribute definitions

*datum_feature_usages*: The *datum_feature_usages* attribute specifies a set of zero or more **Datum_feature_usage_in_datum**s. Each of the **Datum_feature_usage_in_datum**s in this set corresponds to the usage of a datum feature in establishing the datum.

### 4.3 Simple_datum

A **Simple_datum** is a type of **Datum** that corresponds to a datum that is established from exactly one datum feature.

#### Constraints

WR1: Each **Simple_datum** shall be specified as the *used_datum* by at least one **Datum_usage_in_datum_system**.

NOTE—WR1 corresponds to the assertion that each simple datum shall be used in at least one datum system.

WR2: Each **Simple_datum** shall specify exactly one **Datum_feature_usage_in_simple_datum** as its *datum_feature_usages*.

NOTE—WR2 corresponds to the assertion that each simple datum shall be established from exactly one datum feature.

### 4.4 Common_datum

A **Common_datum** is a type of **Datum** that corresponds to a datum that is established from more than one datum feature.

**Fig. 3:**
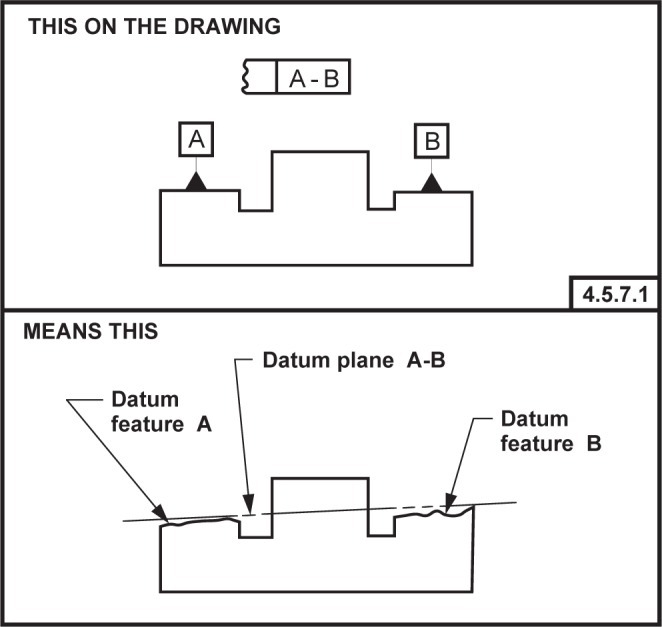
Technical drawing illustrating a common datum. This figure is a reproduction of FIG. 4-20 presented in ASME Y14.5M [4].

NOTE—On technical drawings, a datum that is established from multiple datum features is indicated by placing the identifying letters of the datum features, separated by a dash, within a single compartment in a feature control frame. There is no significance to the order of the datum feature identifying letters within a compartment of the feature control frame.
EXAMPLE—The technical drawing presented in Fig. 3 shows a datum plane that is established from two datum features (datum features A and B).

#### Constraints

WR1: Each **Common_datum** shall be specified as the *used_datum* by at least one **Datum_usage_in_datum_system**.

NOTE—WR1 corresponds to the assertion that each common datum shall be used in at least one datum system.

WR2: Each **Common_datum** shall specify more than one **Datum_feature_usage_in_common_datum** as its *datum_feature_usages*.

NOTE—WR2 corresponds to the assertion that each common datum shall be established from more than one datum feature.

### 4.5 Datum_feature

A **Datum_feature** corresponds to a datum feature (see Sec. 3 of this paper). A **Datum_feature** may be a **Datum_target_set**.

NOTE—The **Datum_feature** entity is based on the **Shape_aspect** entity of STEP Part 41 [11].
NOTE—On technical drawings, a feature is typically identified as a datum feature by means of a datum feature symbol, e.g., 
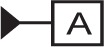
.
NOTE—The concept of datum feature in the DSCDM applies to features that are used to establish one or more datums. Features that may be used as datum features include “partial” features and datum target sets, as well as “complete” and composite features. The concept of datum feature in the DSCDM does not pertain to features in which only a portion of the feature (i.e., a “partial” feature or a datum target set) is used to establish one or more datums. “Partial” and composite features are discussed in Sec. 10.2 of this paper.

#### Attribute definitions

*identification*: The *identification* attribute specifies the string value by which the corresponding datum feature is referred.

NOTE—On technical drawings, each datum feature is referred to by an identifying letter, (e.g., the letter “A” in the datum feature symbol 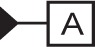).
EXAMPLE—Two datum features are identified in Fig. 3, datum feature A and datum feature B.

#### Inverse attribute definitions

*datum_feature_usages*: The *datum_feature_usages* attribute specifies a set of one or more **Datum_feature_usage_in_datum**s. Each of the **Datum_feature_usage_in_datum**s in this set corresponds to the usage of the datum feature in establishing a datum.

#### Constraints

WR1: There shall be at most one **Datum_feature_usage_in_simple_datum** in the set of **Datum_feature_usage_in_datum**s specified as the *datum_feature_usages*.

NOTE—WR1 corresponds to the assertion that each datum feature shall be used to establish at most one simple datum (a datum that is established from a single datum feature).

### 4.6 Datum_target_set

A **Datum_target_set** is a type of **Datum_feature** that corresponds to a set of one or more datum targets (see Sec. 3 of this paper).

**Fig. 4:**
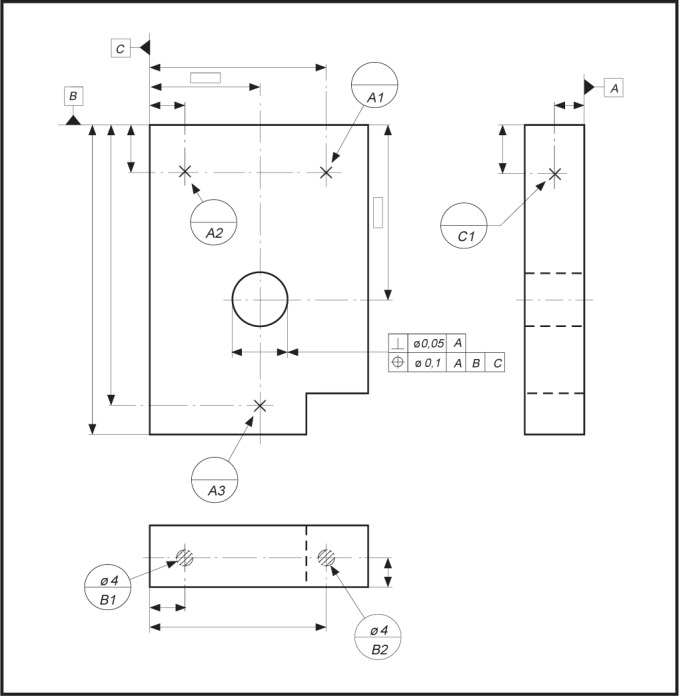
Technical drawing illustrating datum target sets. This figure is a reproduction of a technical drawing presented in Figure 42 of ISO 5459 [6].

EXAMPLE—There are three datum target sets shown in the technical drawing presented in Fig. 4 (datum target sets A, B, and C). The letters in the lower compartment of the datum target frames (e.g., 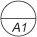) indicate in which datum target sets the associated datum targets are used.

#### Inverse attribute definitions

*datum_target_usages*: The *datum_target_usages* attribute specifies a set of one or more **Datum_target_usage_in_datum_target_set**s. Each of the **Datum_target_usage_in_datum_target_set**s in this set corresponds to the usage of a datum target in the datum target set.

### 4.7 Datum_target

A **Datum_target** corresponds to a datum target (see Sec. 3 of this paper).

NOTE—The **Datum_target** entity is based on the **Shape_aspect** entity of STEP Part 41 [11].
NOTE—Datum targets are typically used in situations where it is inappropriate to specify an entire surface as a datum feature.
EXAMPLE—There are six datum targets shown in Fig. 4. Four of these datum targets are datum target points, each of which is represented by an ×. The other two datum targets are datum target areas, each of which is represented by a cross-hatched circular area.

#### Inverse attribute definitions

*datum_target_usage*s: The *datum_target_usages* attribute specifies a set of one or more **Datum_target_usage_in_datum_target_set**s. Each of the **Datum_target_usage_in_datum_target_set**s in this set corresponds to the usage of the datum target in a datum target set.

### 4.8 Datum_usage_in_datum_system

A **Datum_usage_in_datum_system** corresponds to the usage of a datum in a datum system.

NOTE—The **Datum_usage_in_datum_system** entity is based on the **Shape_aspect_relationship** entity of STEP Part 41 [11].

#### Attribute definitions

*comprised_datum_system*: The *comprised_datum_system* attribute specifies the **Datum_system** that corresponds to the datum system that is either partially or wholly comprised of the corresponding datum.

*used_datum*: The *used_datum* attribute specifies the **Datum** that corresponds to the datum that is used in the corresponding datum system.

#### Inverse attribute definitions

*precedence_assignment*: The *precedence_assignment* attribute specifies the **Datum_precedence_assignment** that corresponds to the specification of the order in which the datum is established within the datum system.

#### Constraints

UR1: The combination of *comprised_datum_system* and *used_datum* shall be unique within a population of **Datum_usage_in_datum_system**.

NOTE—UR1 corresponds to the assertion that each datum shall not be used more than once in any one datum system.

WR1: The **Datum** specified as the *used_datum* shall either be a **Common_datum** or **Simple_datum**.

NOTE–WR1 corresponds to the assertion that each datum that is used in a datum system shall be established from one or more datum features.

### 4.9 Datum_feature_usage_in_datum_system

A **Datum_feature_usage_in_datum_system** corresponds to the usage of a datum feature in establishing a datum system.

NOTE—The **Datum_feature_usage_in_datum_system** entity is based on the **Shape_aspect_relationship** entity of STEP Part 41 [11].
NOTE—The relationship between a **Datum_feature** and a **Datum_system** is indirectly established with a **Datum_feature_usage_in_datum**, a **Datum**, and a **Datum_usage_in_datum_system**. Therefore, a **Datum_feature_usage_in_datum_system** should not be used unless it is necessary to indicate the application of either the *least material requirement or the maximum material principle* (see Sec. 3 of this paper) to a datum feature within the context of a datum system. In essence, a **Datum_feature_usage_in_datum_system** corresponds to a datum feature in the context of a datum system.

#### Attribute definitions

*established_datum_system*: The *established_datum_system* attribute specifies the **Datum_system** that corresponds to the datum system that is established from the corresponding datum feature.

*used_datum_feature*: The *used_datum_feature* attribute specifies the **Datum_feature** that corresponds to the datum feature that is used to establish the corresponding datum system.

#### Inverse attribute definitions

*applied_material_condition_property*: The *applied_material_condition_property* attribute specifies the **Datum_feature_material_condition_property** that corresponds to the specification of a material condition property (i.e., *least material requirement or the maximum material principle*) that is applied to the datum feature in the context of the datum system.

#### Constraints

WR1: The **Datum_feature** specified as the *used_datum_feature* shall be specified as the *used_datum_feature* by a **Datum_feature_usage_in_datum** that specifies a **Datum** as the *established_datum*, and that **Datum** shall be specified as the *used_datum* by a **Datum_usage_in_datum_system** that specifies the same **Datum_system** as the *comprised_datum_system*, as is specified as the *established_datum_system* by the **Datum_feature_usage_in_datum_system**.

NOTE—WR1 corresponds to the assertion that the datum feature shall be used to establish a datum that is used in the datum system.

### 4.10 Datum_feature_usage_in_datum

A **Datum_feature_usage_in_datum** corresponds to the usage of a datum feature in establishing a datum. A **Datum_feature_usage_in_datum** is either a **Datum_feature_usage_in_simple_datum** or a **Datum_feature_usage_in_common_datum**.

NOTE—The **Datum_feature_usage_in_datum** entity is based on the **Shape_aspect_relationship** entity of STEP Part 41 [11].

#### Attribute definitions

*established_datum*: The *established_datum* attribute specifies the **Datum** that corresponds to the datum that is established from the corresponding datum feature.

*used_datum_feature*: The *used_datum_feature* attribute specifies the **Datum_feature** that corresponds to the datum feature that is used to establish the corresponding datum.

### 4.11 Datum_feature_usage_in_simple_datum

A **Datum_feature_usage_in_simple_datum** is a type of **Datum_feature_usage_in_datum** that corresponds to the usage of a datum feature in establishing a datum that is established from exactly one datum feature.

#### Attribute definitions

*established_datum*: The *established_datum* attribute specifies the **Simple_datum** that corresponds to the datum that is established from the corresponding datum feature.

NOTE—“The corresponding datum feature” refers to the datum feature that corresponds to the **Datum_feature** specified by the inherited *used_datum_feature attribute*.

### 4.12 Datum_feature_usage_in_common_datum

A **Datum_feature_usage_in_common_datum** is a type of **Datum_feature_usage_in_datum** that corresponds to the usage of a datum feature in establishing a datum that is established from more than one datum feature.

#### Attribute definitions

*established_datum*: The *established_datum* attribute specifies the **Common_datum** that corresponds to the datum that is established, in part, from the corresponding datum feature.

NOTE—“The corresponding datum feature” refers to the datum feature that corresponds to the **Datum_feature** specified by the inherited *used_datum_feature* attribute.

### 4.13 Datum_target_usage_in_datum_target_set

A **Datum_target_usage_in_datum_target_set** corresponds to the usage of a datum target in a set of datum targets.

NOTE—The **Datum_target_usage_in_datum_target_set** entity is based on the **Shape_aspect_relationship** entity of STEP Part 41 [11].
NOTE—On technical drawings, the usage of a datum target in a datum target set is indicated with a datum target frame, e.g., the 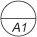 symbol in Fig. 4. The letter in the lower compartment of a datum target frame identifies the datum target set in which the associated datum target is used, and the number (datum target number) in the lower compartment is an integer value by which the associated datum target is identified within the datum target set.
EXAMPLE—The 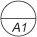 datum target frame in Fig. 4 indicates that the datum target pointed to by the connected leader line is used within datum target set A.

#### Attribute definitions

*comprised_datum_target_set*: The *comprised_datum_target_set* attribute specifies the **Datum_target_set** that corresponds to the datum target set that is either partially or wholly comprised of the corresponding datum target.

*datum_target_number*: The *datum_target_number* attribute specifies the integer value by which the corresponding datum target is identified within the corresponding datum target set.

NOTE—Datum target numbers are described in 7.1.1 of ISO 5459 [6].
EXAMPLE—The datum target number “1” in the 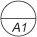 datum target frame of Fig. 4 is the integer value by which the associated datum target is identified within datum target set A.

*used_datum_target*: The *used_datum_target* attribute specifies the **Datum_target** that corresponds to a datum target that is used in the corresponding datum target set.

#### Constraints

UR1: The combination of *used_datum_target* and *defined_datum_target_set* shall be unique within a population of **Datum_target_usage_in_datum_target_set**.

NOTE—UR1 corresponds to the assertion that each datum target shall not be used in any one datum target set more than once.

UR2: The combination of *datum_target_number* and *defined_datum_target_set* shall be unique within a population of **Datum_target_usage_in_datum_target_set**.

NOTE—UR2 corresponds to the assertion that within a datum target set each datum target shall be identified by a unique datum target number.

### 4.14 Datum_system_definition

A **Datum_system_definition** corresponds to the specification of the characteristics of a datum system. These characteristics include the order in which the datums are established within the datum system and any material condition properties (i.e., *least material requirement or maximum material principle*) that are explicitly applied to datum features within the context of the datum system. A **Datum_system_definition** shall either be a **Datum_system_definition_with_material_conditions** or a **Datum_system_definition_without_material_conditions**.

NOTE—The **Datum_system_definition** entity is based on the **Property_definition** entity of STEP Part 41 [11].
NOTE—On technical drawings, the characteristics of a datum system are typically specified in a feature control frame.

#### Attribute definitions

*defined_datum_system*: The *defined_datum_system* attribute specifies the **Datum_system** that corresponds to the datum system the characteristics of which are specified.

*assigned_datum_precedences*: The *assigned_datum_precedences* attribute specifies a set of one to three **Datum_precedence_assignment**s. Each of the **Datum_precedence_assignment**s in this set corresponds to the specification of the order in which a datum is established within the datum system.

#### Constraints

WR1: Each **Datum_precedence_assignment** within the set of **Datum_precedence_assignment**s specified as the *assigned_datum_precedences* shall specify as its *assigned_to* a **Datum_usage_in_datum_system** that specifies as its *comprised_datum_system* the same **Datum_system** as specified as the *defined_datum_system*.

NOTE—WR1 corresponds to the assertion that each datum system specification shall only specify the precedence of datums used in the datum system that the specification characterizes.

WR2: A **Datum_precedence_assignment** that has a *name* of TERTIARY shall not exist within the set of **Datum_precedence_assignment**s specified as the *assigned_datum_precedences* unless a **Datum_precedence_assignment** exists within that set that has a *name* of SECONDARY.

NOTE—WR2 corresponds to the assertion that each datum system specification that specifies a tertiary datum shall also specify a secondary datum.

WR3: A **Datum_precedence_assignment** that has a *name* of SECONDARY shall not exist within the set of **Datum_precedence_assignment**s specified as the *assigned_datum_precedences* unless a **Datum_precedence_assignment** exists within that set that has a *name* of PRIMARY.

NOTE—WR3 corresponds to the assertion that each datum system specification that specifies a secondary datum shall also specify a primary datum.

WR4: Each **Datum_system_definition** shall be specified as the *referenced_datum_system_definition* by at least one **Geometric_tolerance_with_specified_datum_systrem** or **Dimension_with_specified_datum_system**.

NOTE—WR4 corresponds to the assertion that each datum system specification shall be referenced by at least one geometric tolerance or dimension.

### 4.15 Datum_system_definition_with_material_conditions

A **Datum_system_definition_with_material_conditions** is a type of **Datum_system_definition** that corresponds to a specification of a datum system that specifies the application of material condition properties (i.e., *least material requirement or maximum material principle*) to one or more datum features within the context of the datum system.

NOTE—On technical drawings, a datum system specification that corresponds to a **Datum_system_definition_with_material_conditions** is specified in a feature control frame that contains either at least one *least material requirement symbol* ( 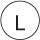) that is preceded immediately by a datum feature letter or at least *one maximum material principle symbol* ( 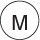) that is preceded immediately by a datum feature letter, e.g., 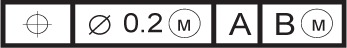 (the 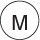 symbol after the tolerance value is associated with the toleranced feature and its representation is not within the scope of this paper).

#### Attribute definitions

*applied_material_condition_properties*: The *applied_material_condition_properties* attribute specifies a set of one or more **Datum_feature_material_condition_property**s. Each of the **Datum_feature_material_condition_property**s in this set corresponds to the specification of a material condition property that is explicitly applied to a datum feature within the context of the datum system.

#### Constraints

WR1: Each **Datum_feature_material_condition_property** within the set of **Datum_feature_material_condition_property**s specified as the *applied_material_condition_properties* shall specify as its *applied_to* a **Datum_feature_usage_in_datum_system** that specifies as its *established_datum_system* the same **Datum_system** as specified as the *defined_datum_system*.

NOTE—WR1 corresponds to the assertion that each datum system specification shall only specify material condition properties for datum features used to establish the datum system that the specification characterizes.
NOTE—The *defined_datum_system* attribute referred to in WR1 is inherited from the **Datum_system_definition** entity of which this entity is a subtype.

### 4.16 Datum_system_definition_without_material_conditions

A **Datum_system_definition_without_material_conditions** is a type of **Datum_system_definition** that corresponds to a specification of a datum system in which no material condition properties (i.e., *least material requirement or maximum material principle*) are specified.

**Fig. 5:**
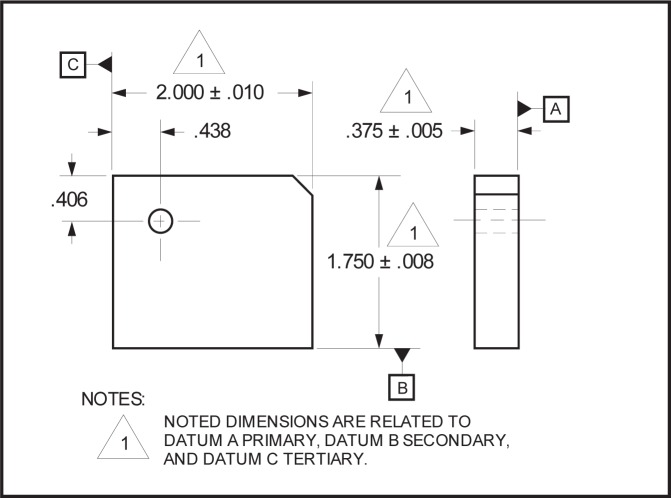
Technical drawing that illustrates the usage of dimensions that reference a datum system specification. The technical drawing is a partial reproduction of Figure 6-46 of *Design Dimensioning and Tolerancing* [13].

NOTE—In technical drawings, a datum system specification that corresponds to a **Datum_system_definition_without_material_conditions** is typically specified in a feature control frame that contains neither a *least material requirement* symbol ( 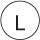) that is immediately preceded by a datum feature letter nor a *maximum material principle* symbol ( 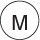) that is immediately preceded by a datum feature letter, e.g., 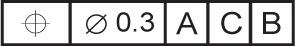.
NOTE—On technical drawings, a datum system specification that corresponds to a **Datum_system_definition_without_material_conditions** could also be specified in a dimension related note; see Fig. 5.

### 4.17 Datum_precedence_assignment

A **Datum_precedence_assignment** corresponds to the specification of the order in which a datum is established within a datum system.

NOTE—The **Datum_precedence_assignment** entity is based on the **Property_definition** entity of STEP Part 41 [11].
NOTE—On technical drawings, the precedence of a datum within a datum system is typically specified in a feature control frame. The location of the compartment containing the letter(s) corresponding to the datum feature(s) from which the datum is established indicates the assigned precedence. The compartment for the primary datum (if it exists) is immediately to the right of the compartment containing the tolerance value. The compartment for the secondary datum (if it exists) is immediately to the right of the compartment for the primary datum. Lastly, the compartment for the tertiary datum (if it exists) is immediately to the right of the compartment for the secondary datum.
EXAMPLE—Figure 2 (b) contains a feature control frame that specifies a datum system in which datum A is the primary datum, datum B is the secondary datum, and datum C is the tertiary datum. Similarly, Fig. 2 (c) contains a feature control frame that specifies a datum system in which datum A is the primary datum, datum C is the secondary datum, and datum B is the tertiary datum.

#### Attribute definitions

*assigned_to:* The *assigned_to* attribute specifies a **Datum_usage_in_datum_system**. In essence, the **Datum_usage_in_datum_system** corresponds to the datum within the context of the datum system to which the datum precedence is assigned.

**Fig. 6:**
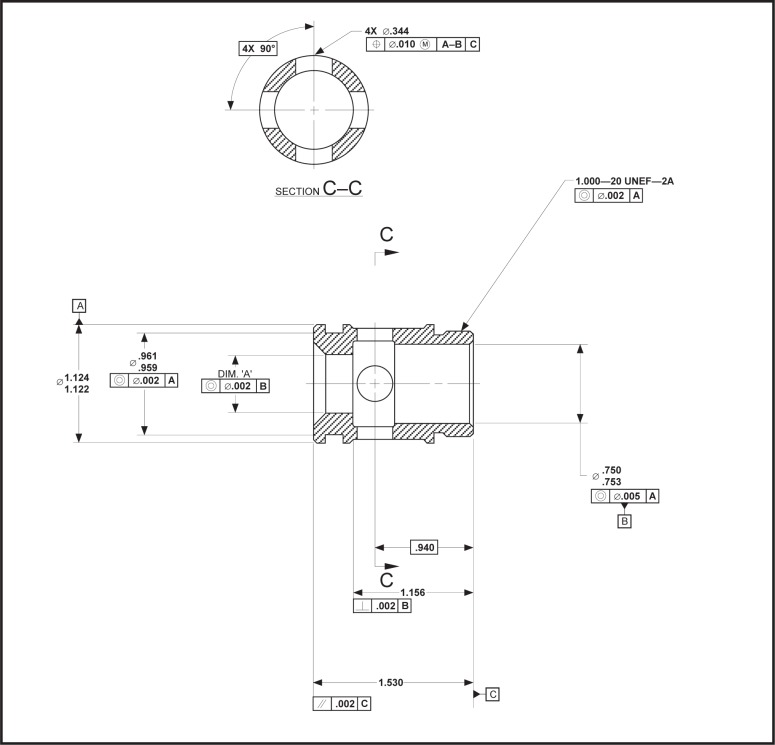
Technical drawing of a hydraulic valve. This technical drawing is a partial reproduction of a drawing presented on page 308 in *Geometric Dimensioning and Tolerancing* [14].

NOTE—A datum within the context of one datum system may be assigned one precedence, e.g., primary, and the same datum within the context of another datum system may be assigned another precedence, e.g., secondary.
EXAMPLE—In Fig. 6, datum feature C (the end surface of the part that is shown on the right side) is used to establish a datum plane. The top-most feature control frame asserts that this datum is the secondary datum within the context of one datum system. Furthermore, the bottom-most feature control frame asserts that this datum is the primary datum within the context of another datum system.

*name:* The *name* attribute specifies the value of the assigned datum precedence. Valid values for the *name* are PRIMARY, SECONDARY, and TERTIARY.

#### Inverse attribute definitions

*associate_datum_system_definition:* The *associate_datum_system_definition* attribute specifies the **Datum_system_definition** that corresponds to the datum system specification to which the datum precedence is associated.

#### Constraints

UR1: The combination of *name* and *associate_datum_system_definition* shall be unique within a population of **Datum_precedence_assignment**s.

NOTE—UR1 corresponds to the assertion that no two datums of a datum system shall have the same precedence.

### 4.18 Datum_feature_material_condition_property

A **Datum_feature_material_condition_property** corresponds to the specification of a material condition property (i.e., *least material requirement* or *maximum material principle*) that is explicitly applied to a datum feature within the context of a datum system.

NOTE—The **Datum_feature_material_condition_property** entity is based on the **Property_definition** entity of STEP Part 41 [11].

#### Attribute definitions

*applied_to:* The *applied_to* attribute specifies a **Datum_feature_usage_in_datum_system**. In essence, the **Datum_feature_usage_in_datum_system** corresponds to the datum feature within the context of the datum system to which the material condition property is applied.

NOTE—A datum feature within the context of one datum system may have one material condition property applied, e.g., *least material requirement*, and the same datum feature within the context of another datum system may have another material condition property applied, e.g., *maximum material principle*.

*name*: The *name* attribute specifies the value by which the material condition property is known. Valid values for the *name* are LEAST_MATERIAL_REQUIREMENT and MAXIMUM_MATERIAL_PRINCIPLE (see Sec. 3 of this paper).

NOTE—A **Datum_feature_material_condition_property** that has a *name* of LEAST_MATERIAL_REQUIREMENT corresponds to a datum feature letter followed by the 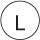 symbol in a feature control frame of a technical drawing, e.g., 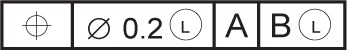 (the 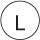 symbol after the tolerance value is associated with the toleranced feature and its representation is not within the scope of this paper).
NOTE—A **Datum_feature_material_condition_property** that has a *name* of MAXIMUM_MATERIAL_PRINCIPLE corresponds to a datum feature letter followed by the 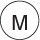 symbol in a feature control frame of a technical drawing, e.g., 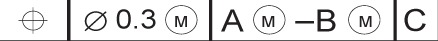 (the 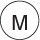 symbol after the tolerance value is associated with the toleranced feature and its representation is not within the scope of this paper).
NOTE—It shall be understood that the *regardless of feature size principle* (see Sec. 3 of this paper) shall be in effect in cases where the datum feature is a feature of size (see Sec. 3 of this paper) and a **Datum_feature_material_condition_property** is not specified.

#### Inverse attribute definitions

*associate_datum_system_definition*: The *associate_datum_system_definition* attribute specifies the **Datum_system_definition_with_material_conditions** that corresponds to the datum system specification to which the material condition property is associated.

### 4.19 Geomsetric_tolerance_with_specified_datum_system

The **Geometric_tolerance_with_specified_datum_system** entity is not completely defined here, as it is not within the scope of this paper. However, the *referenced_datum_system_definition* attribute of this entity is defined to illustrate how the DSCDM could be tied into a larger GD&T data model.

NOTE—The **Geometric_tolerance_with_specified_datum_system** entity is based on the **Property_definition** entity of STEP Part 41 [11].

#### Attribute definitions

*referenced_datum_system_definition*: The *referenced_datum_system_definition* attribute specifies the **Datum_system_definition** that corresponds to the datum system specification that is referenced by the geometric tolerance.

### 4.20 Dimension_with_specified_datum_system

The **Dimension_with_specified_datum_system** entity is not completely defined here, as it is not within the scope of this paper. However, the *referenced_datum_system_definition* attribute of this entity is defined to illustrate how the DSCDM could be tied into a larger GD&T data model.

NOTE—The **Dimension_with_specified_datum_system** entity is based on the **Property_definition** entity of STEP Part 41 [11].
NOTE—While the data modeled with the **Datum_system_definition** entity is associated almost exclusively with geometric tolerances, clause 4.4 of ASME Y 14.5M [4] describes the usage of this data with linear and angular dimensions. The **Dimension_with_specified_datum_system** entity is shown in Fig. 1 to illustrate this usage.
EXAMPLE—The three linear dimensions presented in Fig. 5 reference NOTE 1. This note is a specification for a datum system that specifies that the primary datum is established from datum feature A, the secondary datum is established from datum feature B, and the tertiary datum is established from datum feature C.

#### Attribute definitions

*referenced_datum_system_definition*: The *referenced_datum_system_definition* attribute specifies the **Datum_system_definition_without_material_conditions** that corresponds to the datum system specification that is referenced by the dimension.

## 5. Part 47 Datum System Related Model

**Fig. 7:**
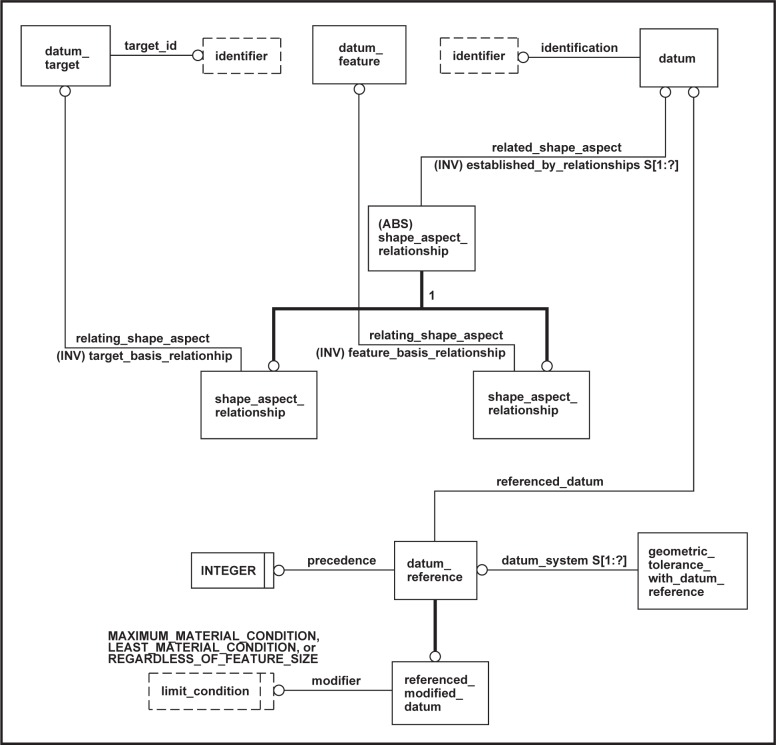
Pseudo EXPRESS-G diagram of datum system related entities of STEP Part 47 [2].

A pseudo EXPRESS-G diagram of the datum system related portion of the Part 47 model is presented in Fig. 7. The term “pseudo” is used because non-standard EXPRESS-G is employed to indicate the constraints placed on the **Datum_target**, **Datum_feature**, and **Datum** entities (e.g., the model shows three **Shape_aspect_relationship** entities). Additionally, the EXPRESS-G diagram does not show that the **Datum_target**, **Datum_feature**, and **Datum** entities are subtypes of the **Shape_aspect** entity of STEP Part 41 [11].

The definitions of the entities shown in Fig. 7 are provided in Tables 1–6. These definitions are taken from STEP Part 47 [2].

NOTE—The actual EXPRESS declarations of these entities have not been included in the definitions given in Tables 1–6, as they are not necessary to the understanding of the concepts presented in this paper.
NOTE—The clause and figure numbers specified within Tables 1–6 are from STEP Part 47 and should not be confused with the clause and figure numbers of this paper.
NOTE—The definition of the **Geometric_tolerance** entity of which the **Geometric_tolerance_with_datum_reference** entity is a subtype is not shown here, as it is not within the scope of this paper.

## 6. The DSCDM Compared to the Part 47 Model

This section discusses the differences between the DSCDM and portions of the model presented in STEP Part 47 [2] that are related to datums. Inasmuch as STEP Part 47 is an integrated generic resource, it should not necessarily be as specialized as the DSCDM. Still, it is useful to make certain comparisons between these two models to observe how they differ in representing the datum related concepts presented in some of the GD&T drawing-based standards. It is particularly useful to note cases that cannot be clearly represented with the Part 47 model and in which the deficiency is not due to the generic nature of the Part 47 model.

NOTE—The STEP architecture is such that STEP application protocols may specialize entities from the STEP integrated generic resources. However, deficiencies in entities of the type mentioned above will only be passed on to the STEP application protocols that incorporate them.
EXAMPLE—A STEP application protocol that incorporates the **Datum_feature** entity from STEP Part 47 will not be able to support multiple use datum features (see Sec. 6.3).

### 6.1 Datum_system and Datum_system_definition

One of the main differences between the Part 47 model and the DSCDM is that the Part 47 model has no entities that are equivalent to the **Datum_system** and **Datum_system_definition** entities of the DSCDM. Two independent comments were submitted against the STEP Part 47 DIS [15] document indicating that the concept of datum precedence only made sense within the context of a datum system. Additionally, one of those comments also indicated that the modifier for the **Datum_reference** only made sense in the context of a datum system. Concurring with those comments, the **Datum_system** and **Datum_system_definition** entities were incorporated within the DSCDM.

### 6.2 Ambiguous Datum Feature Identification

In the DSCDM the *identification* attribute is on the **Datum_feature** entity; in contrast, in the Part 47 model the *identification* attribute is on the **Datum** entity. In practice, it is the datum feature to which an identifier is assigned. ASME Y14.5M [4] states, “Each datum feature of a part requiring identification shall be assigned a different letter.” Furthermore, datums associated with datum systems are identified by the datum features from which they are established. In cases in which a datum is established from a single datum feature, the location of the *identification* attribute may seem moot, because if the *identification* attribute is placed on the **Datum** entity, the name of the associated datum feature could easily be derived. However, in cases in which a datum is established from more than one datum feature, the Part 47 model produces ambiguous results because it is impossible to determine the name of the datum features from the value of the *identification* attribute on a **Datum**. The DSCDM does not have this ambiguity, as the *identification* attribute is on the **Datum_feature** entity.

EXAMPLE—The 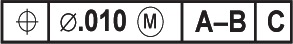 feature control frame of the position tolerance in Fig. 6 indicates that the primary datum is established from datum feature A in conjunction with datum feature B. If the Part 47 model was used to describe this requirement, the *identification* attribute of the **Datum** entity would have a value of “A–B”. However, it would be unclear as to which datum feature is identified as A and which datum feature is identified as B.
NOTE—As the **Datum** and **Datum_feature** entities in the Part 47 model are subtypes of the **Shape_aspect** entity of STEP Part 41 [11], they both inherit a *name* attribute. However, as a **Datum_feature** corresponds to an actual feature of a part it is likely that the *name* attribute will not be available for the datum feature identifying letter because it will likely be used for another purpose (e.g., the name given to a feature prior to its promotion to a datum feature). Furthermore, as datums are identified solely for GD&T purposes it is likely that the inherited *name* attribute on the **Datum** entity would be available, thereby making the *identification* attribute on the **Datum** entity not only misplaced but redundant.

### 6.3 Multiple Use Datum Features

The Part 47 model fails to account for the fact that a datum feature may be used to establish multiple datums, whereas the DSCDM does account for this fact. In the Part 47 model, the *feature_basis_relationship* attribute and WR1 on the **Datum_feature** entity specify that a **Datum_feature** shall be related to exactly one **Datum**. On the other hand, in the DSCDM the inverse *datum_feature_usages* attribute on the **Datum_feature** entity constrains the number of **Datum**s that shall be established from a **Datum_feature** to one or more.

EXAMPLE—The technical drawing presented in Fig. 6 illustrates how a datum feature may be used to establish multiple datums. This figure shows that datum feature A (the outer most cylindrical surface) is used to establish the primary datum (a center axis) of the datum system specified by three concentricity tolerances (e.g., 
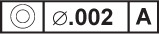
). Also, Fig. 6 shows that datum feature B (the inner hole surface on the right side) is used to establish the primary datum (another center axis) of the datum system specified by a concentricity tolerance and a perpendicularity tolerance (i.e., 
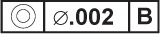
, and 
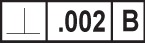
). Furthermore, both datum features A and B are used once again to establish the primary datum (yet another center axis) of the datum system specified by the position tolerance (i.e., 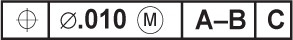). As the Part 47 model limits the number of datums that may be established from a datum feature to one, this situation cannot be represented with the Part 47 model.

### 6.4 Multiple Use Datum Targets

**Fig. 8:**
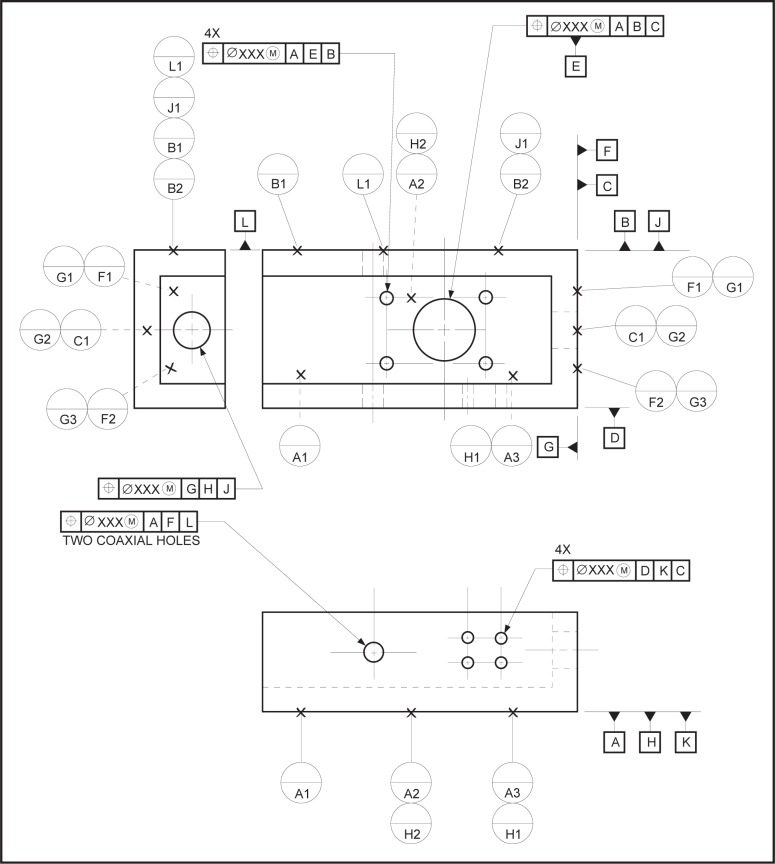
Technical drawing illustrating multiple use of datum targets. This technical drawing is a reproduction of a drawing presented on page 265 in *Geo-metrics IIIm* [8].

The Part 47 model fails to account for the fact that a datum target may be used to establish multiple datums, whereas the DSCDM does account for this fact. In the Part 47 model the *target_basis_relationship* attribute and WR1 on the **Datum_target** entity specify that a **Datum_target** shall be related to exactly one **Datum**. On the other hand, the DSCDM does not specify a direct relationship between a **Datum_target** and a **Datum**. Instead, in the DSCDM the relationship between a **Datum_target** and a **Datum** is specified indirectly via the **Datum_target_set** entity and the two relationship entities **Datum_target_usage_in_datum_target_set** and **Datum_feature_usage_in_datum**. In the DSCDM the constraints on **Datum_target** and **Datum_feature** correspond to the assertion that a datum target shall be used in at least one datum target set and because a datum target set is a type of datum feature, the datum target set shall be used to establish at least one datum. The technical drawing presented in Fig. 8 shows nine datum targets (datum target points are indicated by the × symbols), six of which are used to establish multiple datums.

EXAMPLE—The datum target point in Fig. 8 that is connected to the 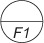 and 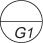 datum target frames is associated with two datum target sets, F and G, each of which is used to establish a separate datum. As the Part 47 model limits the number of datums that may be established from a datum target to one, this situation cannot be represented with the Part 47 model.

### 6.5 Datum Target Sets

In the DSCDM, a **Datum_target_set** is a type of **Datum_feature**. The Part 47 model has no entity that is equivalent to the **Datum_target_set** entity. Furthermore, the Part 47 model prevents the **Datum_feature** entity or a specialization (i.e., subtype) of it to serve as a set of datum targets. That is, in the Part 47 model the attributes *target_basis_relationship* and *feature_basis_relationship* on the **Datum_target** and **Datum_feature** entities, respectively, in association with WR1 on each of these entities, prevent a **Datum_target** from being related to a **Datum_feature** via a **Shape_aspect_relationship**.

NOTE—On technical drawings, datum target frames are used to group datum targets into datum target sets.
EXAMPLE—In the technical drawing presented in Fig. 4, the three datum target points (× symbols are used to indicate datum target points) that are connected to the 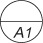, 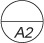 and 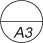 datum target frames make up datum target set A. Additionally, the two datum target areas (hatched regions are used to indicate datum target areas) that are connected to the 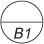 and 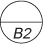 datum target frames constitute datum target set B. Also, the datum target point that is connected to the 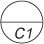 datum target frame makes up datum target set C.

### 6.6 Multiple Datum Target Numbers

The Part 47 model fails to account for the fact that multiple datum target numbers may be associated with a datum target, whereas the DSCDM does account for this fact. In the Part 47 model, the definition for the *target_id* attribute on the **Datum_target** entity indicates that the use of this attribute is to associate a datum target number with a datum target. However, the placement of this attribute on the **Datum_target** entity only allows a single datum target number to be associated with a datum target, which is not surprising as the Part 47 model only allows a datum target to be associated with a single datum. On the other hand, in the DSCDM the placement of the *datum_target_number* attribute on the **Datum_target_usage_in_datum_target_set** entity permits a different datum target number to be assigned to each usage of a datum target in a datum target set.

NOTE—On technical drawings, datum target frames are used to group datum targets into datum target sets. Additionally, datum target frames specify datum target numbers by which the datum targets are identified within the datum target sets.
The technical drawing presented in Fig. 8 shows several instances in which a datum target is identified by multiple datum target numbers, one for each usage of a datum target in a datum target set.
EXAMPLE—The datum target point in Fig. 8 that is connected to the 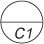 and 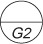 datum target frames is associated with two datum targets sets, C and G. This datum target is identified by a datum target number of “1” when it is associated with datum target set C and is identified by a datum target number of “2” when it is associated with datum target set G.

### 6.7 Composite Datum Features

**Fig. 9:**
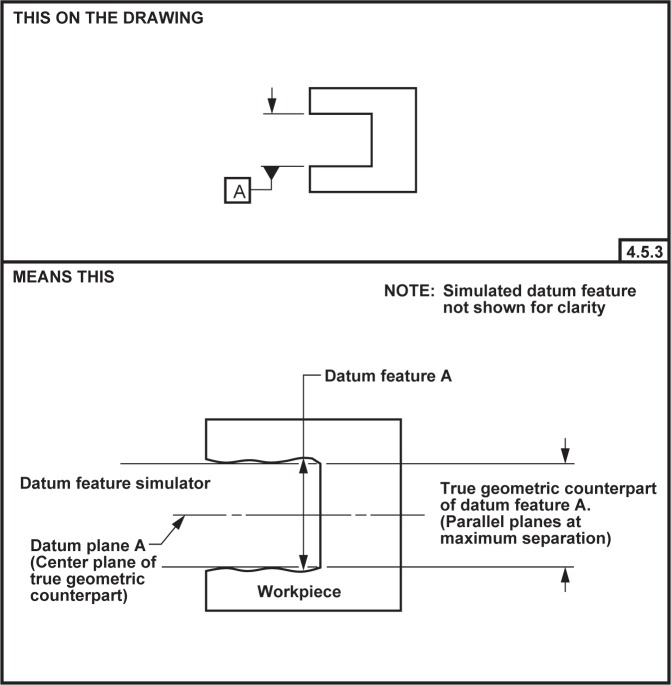
Depiction of a composite datum feature that is composed of two opposing planar features. This figure is a reproduction of FIG. 4-14 of ASME Y14.5M [4].

**Fig. 10:**
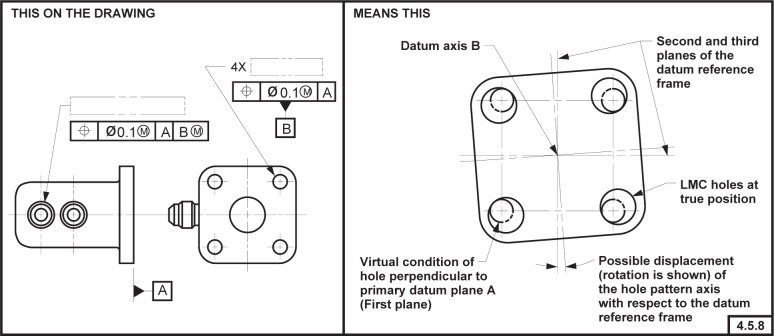
Depiction of a composite datum feature that is composed of four cylindrical features (holes). This figure is a reproduction of FIG. 4-22 of ASME Y14.5M [4].

A composite datum feature is a datum feature that is composed of other features. Figures 9 and 10 depict two examples of composite datum features.

Neither the Part 47 model nor the DSCDM have an explicit entity that corresponds to a composite datum feature. However, it is of interest to examine how composite datum features may be represented using these two models.

**Fig. 11:**
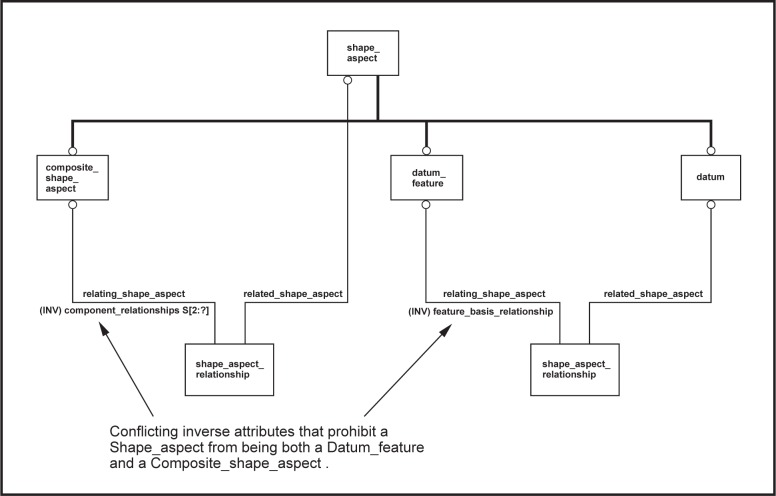
Pseudo EXPRESS-G diagram illustrating why a **Shape_aspect** from STEP 47 cannot be both a **Datum_feature** and a **Composite_shape_aspect**.

The model presented in STEP Part 47 has a **Composite_shape_aspect** entity, the intent of which is to group **Shape_aspects** for a purpose. At first glance this seems like a perfect match—a **Shape_aspect** that is a **Datum_feature** as well as a **Composite_shape_aspect** could represent a composite datum feature. This usage of **Composite_shape_aspect** is even mentioned in a note in clause 4.4.1 of STEP Part 47 [2] (see Table 1 of this paper). Unfortunately, the *feature_basis_relationship* inverse attribute on the **Datum_feature** entity requires that a **Datum_feature** be specified as the *relating_shape_aspect* by exactly one **Shape_aspect_relationship**. Conversely, the *component_relationships* inverse attribute on the **Composite_shape_aspect** entity requires that a **Composite_shape_aspect** be specified as the *relating_shape_aspect* by two or more **Shape_aspect_relationships** (these **Shape_aspect_relationships** relate the **Composite_shape_aspect** with the **Shape_aspects** from which it is composed). This conflict between the two inverse attributes prohibits a **Shape_aspect** from being both a **Datum_feature** and a **Composite_shape_aspect**. Figure 11 is a pseudo EXPRESS-G diagram illustrating this conflict.

Though the DSCDM does not explicitly address the composite datum feature, the DSCDM is constructed such that composite datum features may be addressed by incorporating the DSCDM into a more complete GD&T model that has an entity similar to the **Composite_shape_aspect** entity or by further specializing the **Datum_feature** entity.

NOTE—Appendix C presents one way in which composite datum features may be addressed by a GD&T model that incorporates the DSCDM.

In summary, the DSCDM is designed such that it may be extended to address composite datum features, whereas the Part 47 model is constructed such that even the entities that it indicates should be used to address composite datum features cannot be used for this purpose.

### 6.8 Datum Precedence Assignment

The Part 47 model and the DSCDM differ in two ways with respect to the assignment of datum precedence.

The first difference is that datum precedence is more rigorously defined in the DSCDM than in the Part 47 model. For example, the only constraint associated with the *precedence* attribute in the Part 47 model is that it must be an integer value that is greater than zero. However, the DSCDM has several constraints that ensure that the datums of the datum system have a valid precedence value assigned to them. For example, the UR1 constraint on the **Datum_precedence_assignment** entity corresponds to the assertion that no two datums of a datum system shall have the same precedence value. This lack of rigor in the Part 47 model may be attributed to the fact that STEP Part 47 [2] is a generic integrated resource and as such is not necessarily intended to fully define the requirements.

The second difference is that in the Part 47 model the *precedence* attribute on the **Datum_reference** entity is used to assign a precedence directly to a **Datum**, as opposed to in the DSCDM where the *assigned_to* attribute on the **Datum_precedence_assignment** entity is used to assign a precedence to a **Datum_usage_in_datum_system**. Inasmuch as the **Datum_usage_in_datum_system** entity corresponds to a datum in the context of a datum system, the DSCDM structure asserts that the precedence is assigned to a datum in the context of a datum system.

NOTE—Two of the comments (neither of which were the author’s) submitted against the STEP Part 47 DIS [15] document indicated that the concept of datum precedence only made sense within the context of a datum system. Also, note the wording in clause 2.4.4.2 of STEP Part 41 [11] (see Appendix B of this paper) that describes when a **Property_definition** is applied to a **Shape_aspect_relationship**, “It applies to … an element of the shape in the context of another element of the shape ….” These two details influenced the structure of the DSCDM with respect to what entity is specified by the *assigned_to* attribute of the **Datum_precedence_assignment** entity.

### 6.9 Modifiers and Consistency with ASME Y14.5M

**Fig. 12:**
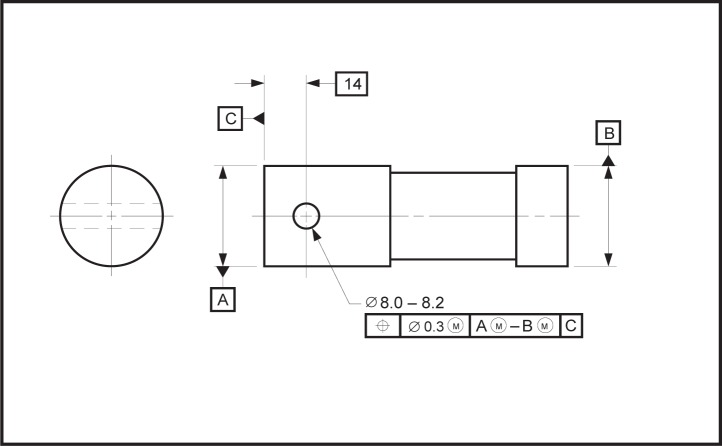
Technical drawing illustrating the application of modifiers to both datum features that establish a common datum. This technical drawing is a reproduction of FIG. 4-19 of ASME Y14.5M [4].

The Part 47 model is inconsistent with ASME Y14.5M [4] with regard to modifiers, whereas the DSCDM is consistent with ASME Y14.5M in this regard. Clause 4.5.7 of ASME Y14.5M states, “Where more than one datum feature is used to establish a single datum, the appropriate datum reference letters and associated modifiers, separated by a dash, are entered in one compartment of a feature control frame.” This statement indicates that modifiers are associated with datum features (see Fig. 12). However, in the Part 47 model the **Referenced_modified_datum** entity is used to associate modifiers with datums, not datum features. On the other hand, in the DSCDM the **Datum_feature_material_condition_property** entity is used to associate modifiers with datum features. (This association is made indirectly through the **Datum_feature_usage_in_datum_system** entity, as the DSCDM asserts that a modifier is applied to a datum feature in the context of a datum system.)

NOTE—The term *datum reference letter* is somewhat of a misnomer, as a *datum reference letter* actually refers to a datum feature.
EXAMPLE—In Fig. 12, the 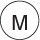 symbol following the letter “A” in the feature control frame associates the *maximum material principle* with datum feature A. Likewise, the 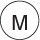 symbol following the letter “B” in the feature control frame associates the *maximum material principle* with datum feature B.

One may argue that if modifiers are directly associated with datums, as in the Part 47 model, they are indirectly associated with the datum features that establish those datums. However, this contrivance fails in cases in which the requirements are such that all the datum features from which a common datum is established are not to be associated with the same modifier.

NOTE—While the DSCDM supports the application of different modifiers to the datum features of a common datum, as is permitted in ASME Y14.5M, the author of this paper has been unable to find examples of this situation in standards or reference books. Therefore, it is believed that occurrences of this case are probably extremely limited.

### 6.10 Dimensions and Datum System Specifications

In the DSCDM the **Dimension_with_specified_datum_system** corresponds to a type of dimension that references a datum system specification. The Part 47 model has no entity that is equivalent to the **Dimension_with_specified_datum_system** entity.

NOTE—The **Dimension_with_specified_datum_system** entity is not completely defined in this paper, as a discussion of dimensions is outside its scope.
NOTE—Clause 4.4 of ASME Y14.5M [4] describes dimensions that reference datum system specifications.
EXAMPLE—The technical drawing presented in Fig. 5 shows three linear dimensions that reference a datum system specification.

### 6.11 Datums Without Datum Features or Datum Targets

The Part 47 model cannot be used to represent datums that are not directly established from datum features or datum targets. This is because the attributes and rules on the **Datum**, **Datum_feature**, and **Datum_target** entities assert that each datum shall be established from one or more datum features or datum targets, that each datum feature shall be used to establish a single datum, and that each datum target shall be used to establish a single datum. In contrast, the DSCDM only requires that the relationship between the datum feature(s) from which a datum is established be specified for those datums that are used to establish a datum system. That is, for a datum not used to establish a datum system (some datums may just be the origin of one or more dimensions), the DSCDM does not require the corresponding **Datum** to be related to a **Datum_feature** via the **Datum_feature_usage_in_datum** entity.

**Fig. 13:**
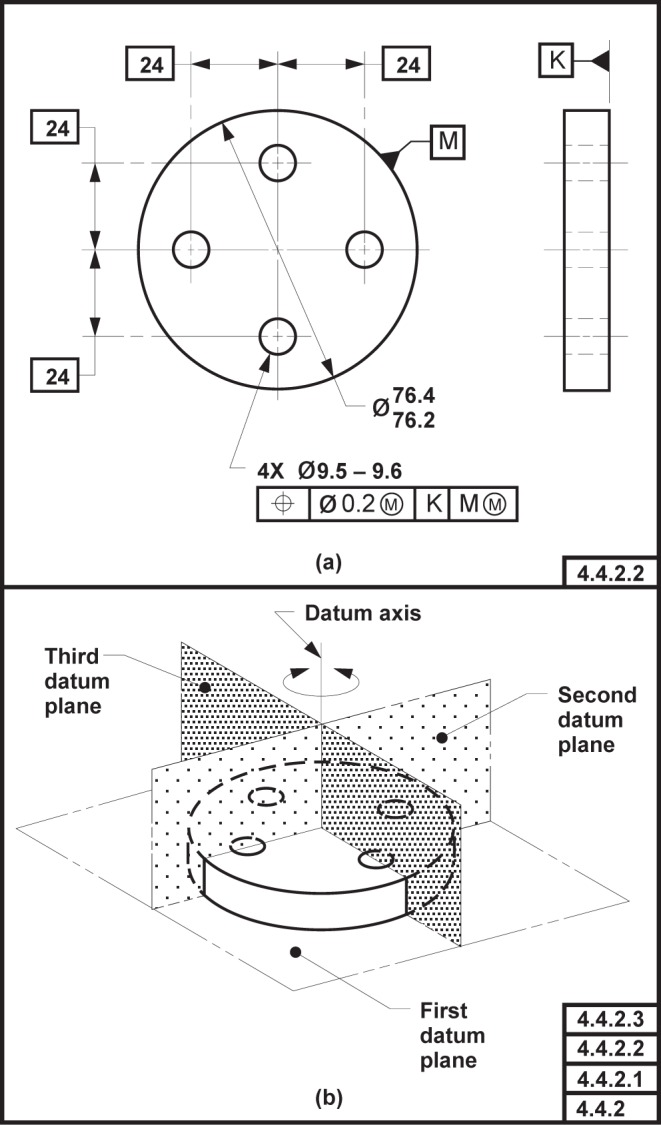
Example of datums not directly established from datum features. This figure is a reproduction of FIG. 4-5 of ASME Y14.5M [4].

EXAMPLE—In Fig. 13(a) the position tolerance references a datum system that is established from two datums: the datum plane [indicated as the “First datum plane” in Fig. 13(b)] that is established from datum feature K and the datum axis [indicated as the “Datum axis” in Fig. 13(b)] that is established from datum feature M. However, two other datum planes (indicated as the “Second datum plane” and “Third datum plane”) also exist and serve as origins for the dimensions that locate the four holes. As there are four datums and only two datum features, the situation shown in Fig. 13 cannot be represented using the Part 47 model (recall the formal propositions on the **Datum**, **Datum_feature**, and **Datum_target** entities in the Part 47 model). However, as the DSCDM does not require a **Datum** to be related to either a **Datum_feature** or a **Datum_system**, this situation can be represented using the DSCDM.
NOTE—Although the datum planes that are labeled “Second datum plane” and “Third datum plane” in Fig. 13 are not part of a datum system, they are part of a datum reference frame. Datum reference frames are not modeled in the DSCDM (datum reference frames are discussed in Appendix D of this paper). However, because the DSCDM does not require a datum to be part of a datum system and does not require a datum to be established from a datum feature, the DSCDM may be incorporated into a larger model that does include datum reference frames.

## 7. Conclusions

This paper has presented a data model (the DSCDM) that covers the concepts of datum systems, datums, datum features, and datum targets. Furthermore, for comparison purposes, this paper has presented the datum related portions of the data model given in STEP Part 47 [2]. In presenting the DSCDM, this paper has used numerous diagrams and examples from existing GD&T drawing-based standards and associated reference books. These diagrams and examples not only provided assistance in explaining the DSCDM but also provided a set of test cases by which the quality of the DSCDM and the Part 47 model were evaluated. This evaluation demonstrated the robustness of the DSCDM and the brittleness of the Part 47 model. Accordingly, it is hoped that the DSCDM may serve as a starting point for the development of more comprehensive GD&T data models, thereby enabling the electronic exchange of GD&T data among the design, manufacturing, and inspection divisions of an enterprise.

## Figures and Tables

**Table 1:** Definition of **Datum** from STEP Part 47 [2].

**4.4.1 Datum**
A **Datum** is a **Shape_aspect** from which dimensions and tolerances are referenced. This **Shape_aspect** may, but need not, coincide with the boundary defining the product. A datum is established by a datum feature, a set of datum targets, or a group of features.
NOTE—The use and application of a group of features to establish a datum is identified in clause 9 of ISO 5459. The group of features is established through the use of **Shape_aspect_relationship** objects. The concept of a group of **Shape_aspect** elements is defined in 4.5.1.
Attribute definitions:
*identification*: the name by which the datum is referred.
*established_by_relationships:* the **Datum_feature**, the set of **Datum_target**s, or the group of derived **Shape_aspect** that establish the **Datum**.
Formal propositions:
WR1: A **Datum** shall be established by either **Datum_feature**s or **Datum_target**s.

**Table 2:** Definition of **Datum_feature** from STEP Part 47 [2].

**4.4.2 Datum_feature**
The **Datum_feature** is an identified **Shape_aspect** on the boundary of the product. One **Datum_feature** may be used to establish a single **Datum**.
Attribute definitions:
SELF\shape_aspect.*product_definitional*: an indicator that the **Datum_feature** is on the physical boundary of the shape that defines the product.
*feature_basis_relationship*: the relationship to the datum that the **Datum_feature** defines; it is achieved through the **Shape_aspect_relationship**.
Formal propositions:
WR1: A **Datum_feature** shall be related to a **Datum**.
WR2: A **Datum_feature** shall lie on the physical boundary of the shape that defines the product.
EXAMPLE 2—Figure 1 illustrates two cases of **Datum_feature**. The **Datum_feature** that is a cylindrical feature establishes the **Datum** identified as A. This **Datum** is the axis of the cylinder. The **Datum** identified as B is established from the **Datum_feature** that is a planar surface of the product. This **Datum** may, but need not, be a plane that is coincident with the **Datum_feature**.
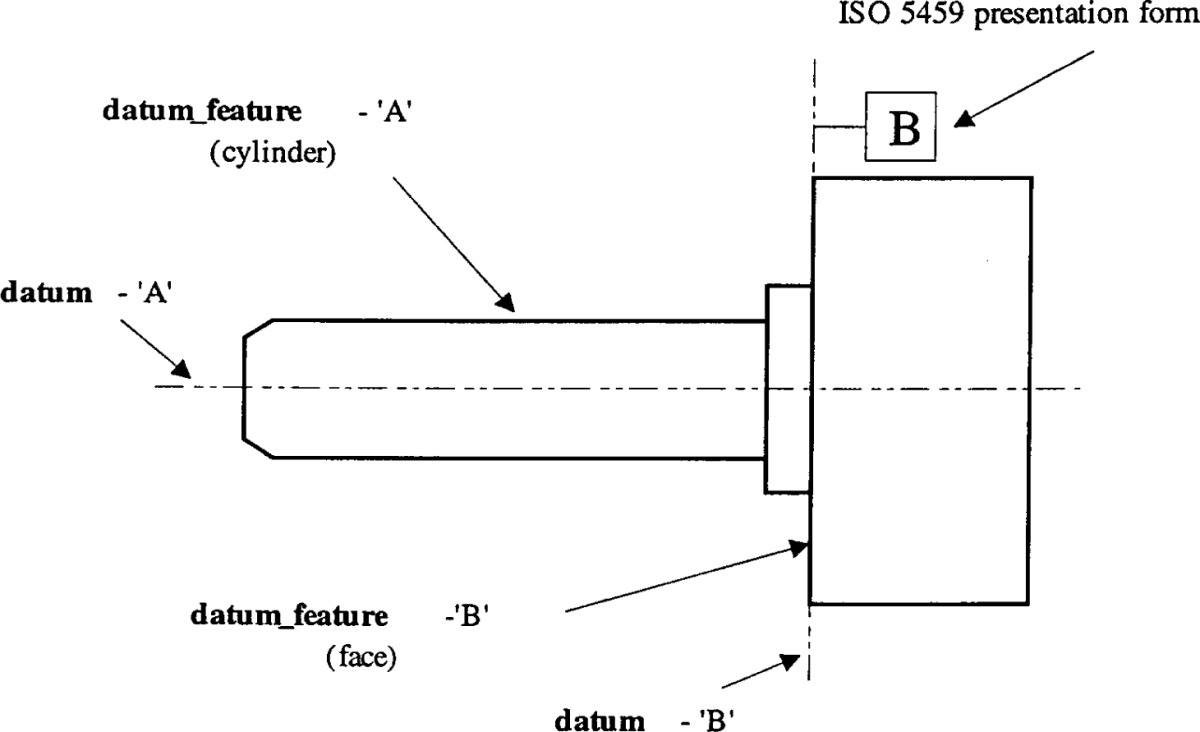
Figure 1 – Examples of datum and datum_feature

**Table 3:** Definition of **Datum_target** from STEP Part 47 [2].

**4.4.3 Datum_target**
The **Datum_target** is a **Shape_aspect** that indicates a datum target on the boundary of a product shape. The **Shape_aspect** may be a point, line, or an area. The **Datum_target** is defined in addition to the **Shape_aspect** elements that define the product shape.
NOTE—The use and application of datum targets is described in clause 7 of ISO 5459.
Attribute definitions:
*target_id*: the name by which the identification of the datum target number is referred.
*target_basis_relationship*: the relationship to the **Datum** that the **Datum_target** defines; it is achieved through the **Shape_aspect_relationship**.
Formal propositions:
WR1: A **Datum_target** shall be related to a **Datum**.
WR2: A **Datum_target** lies on the physical boundary of the shape that defines the product.

**Table 4:** Definition of **Datum_reference** from STEP Part 47 [2].

**4.4.4 Datum_reference**
A **Datum_reference** is the specification of the use of a **Datum**.
EXAMPLE 3—A **Datum** may be used in the definition of multiple **Datum** systems. Each use of the **Datum** would be a **Datum** reference. **Datum** system concepts are described in 6.2 and clause 8 of ISO 5459.
Attribute definitions:
*precedence*: the priority that is assigned to a **Datum** for a specific use.
NOTE—A **Datum** may have multiple and distinct uses and have different precedence for each use.
*referenced_datum*: the **Datum** that participates in a geometrical tolerance of a product feature.
Formal propositions:
WR1: The value of *precedence* shall be greater than zero.

**Table 5:** Definition of **Referenced_modified_datum** from STEP Part 47 [2].

**4.4.5 Referenced_modified_datum**
A **Referenced_modified_datum** is a **Datum_reference** where the referenced datum may vary within the specified limits of size.
NOTES
1—A **Datum** may be modified if the **Datum_feature** that produced it is a product feature which has size characteristics.
2—The use and application of a modified **Datum** are described in clause 8 of ISO 2692.
Attribute definitions:
*modifier*: the **Limit_condition** that is assigned to the **Datum** for a specific use of that **Datum**.

**Table 6:** *Definition of* **Geometric_tolerance_with_datum_reference** from STEP Part 47 [2].

**6.4.4 Geometric_tolerance_with_datum_reference**
A **Geometric_tolerance_with_datum_reference** is a **Geometric_tolerance** that references one or more **Datums** for specifying the tolerance condition of a **Shape_aspect**.
Attribute definitions:
*datum_system*: the datum or combination of datums that define a reference for a **Geometric_tolerance**.
NOTE—This attribute is not equivalent to datum system as defined in clause 3.2 of ISO 5459.

**Fig. A.1:**
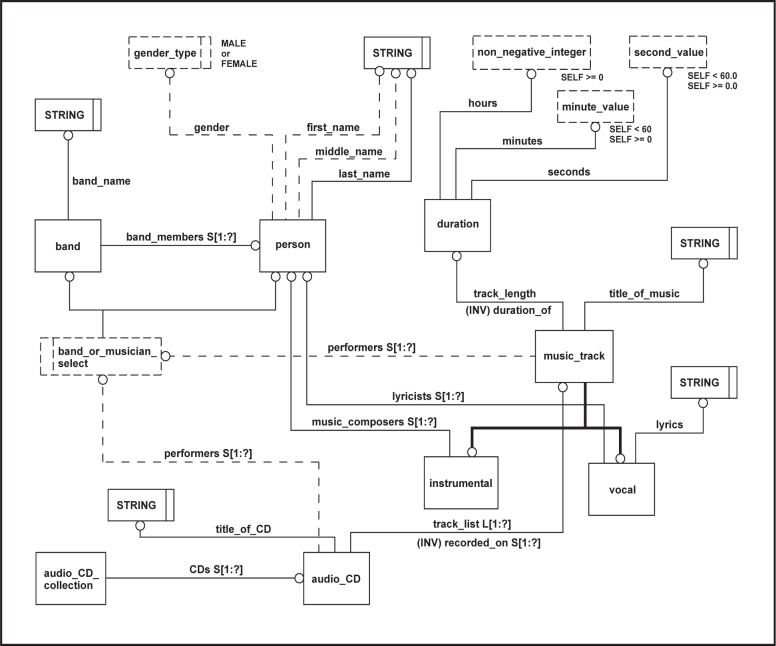
EXPRESS-G model of an audio compact disc collection.

**Fig. A.2:**
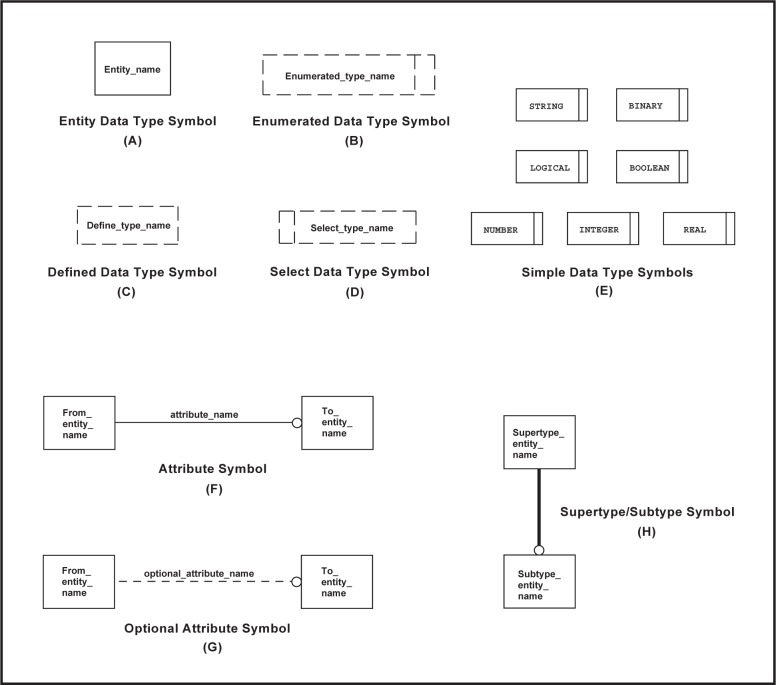
Symbols of the EXPRESS-G notation.

**Fig. B.1:**
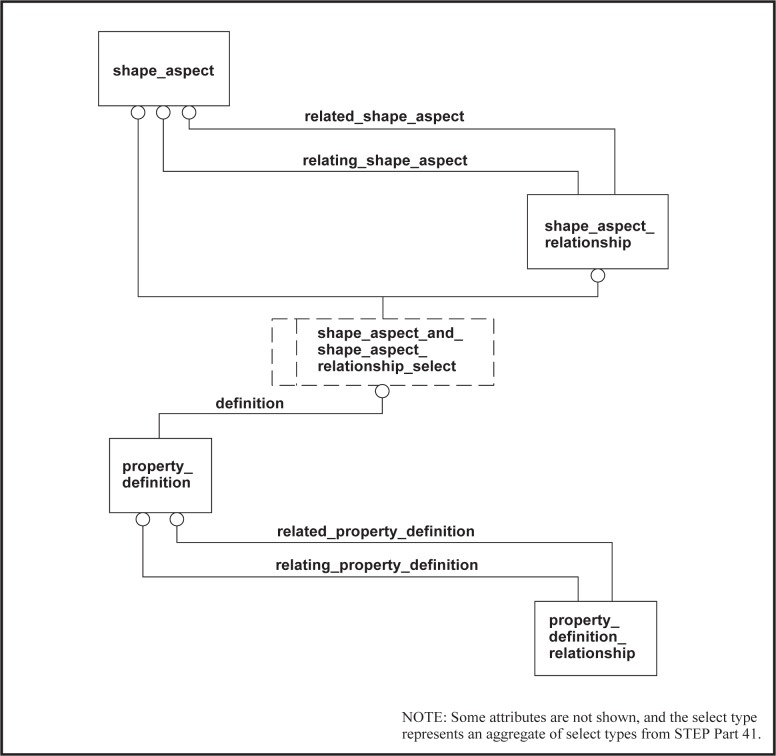
EXPRESS-G diagram illustrating the STEP integrated resources used as a basis for the DSCDM.

**Fig. C.1:**
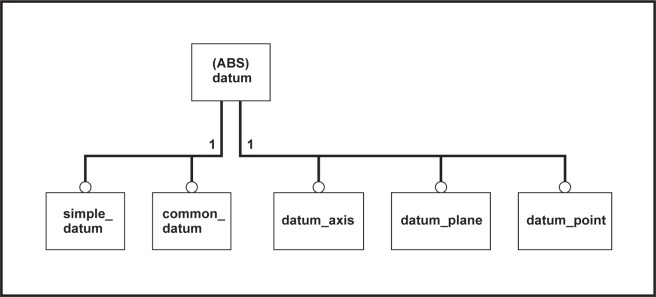
EXPRESS-G diagram showing a possible further specialization of the DSCDM **Datum** entity.

**Fig. C.2:**
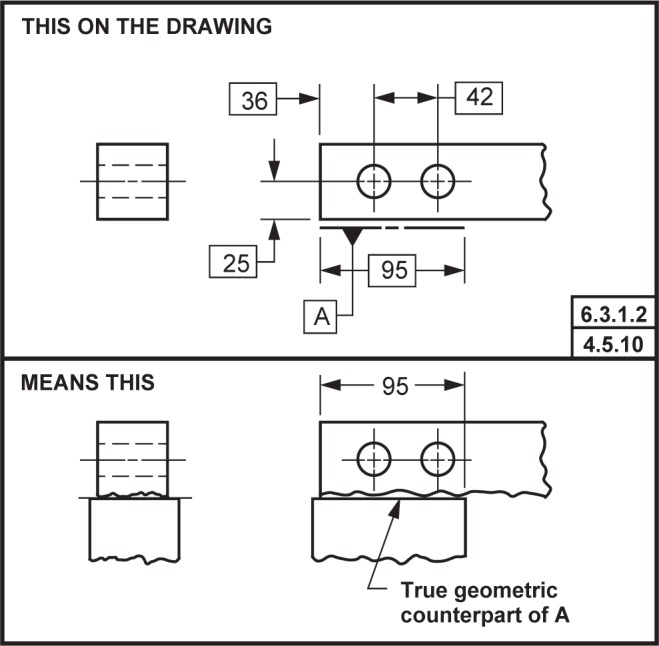
Diagram illustrating a constituent datum feature. This figure is a reproduction of FIG 4-23 presented in ASME Y14.5M [4].

**Fig. C.3:**
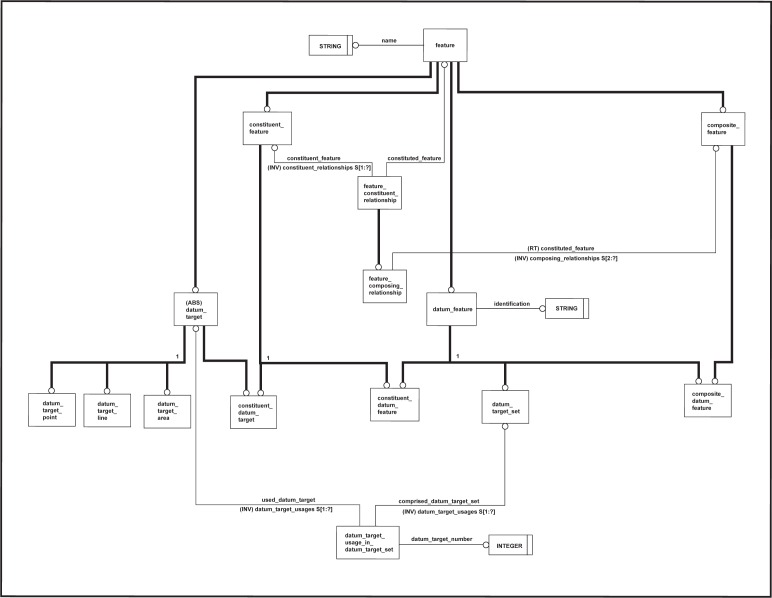
EXPRESS-G diagram showing possible specializations of the DSCDM **Datum_feature** and **Datum_target** entities.

**Fig. C.4:**
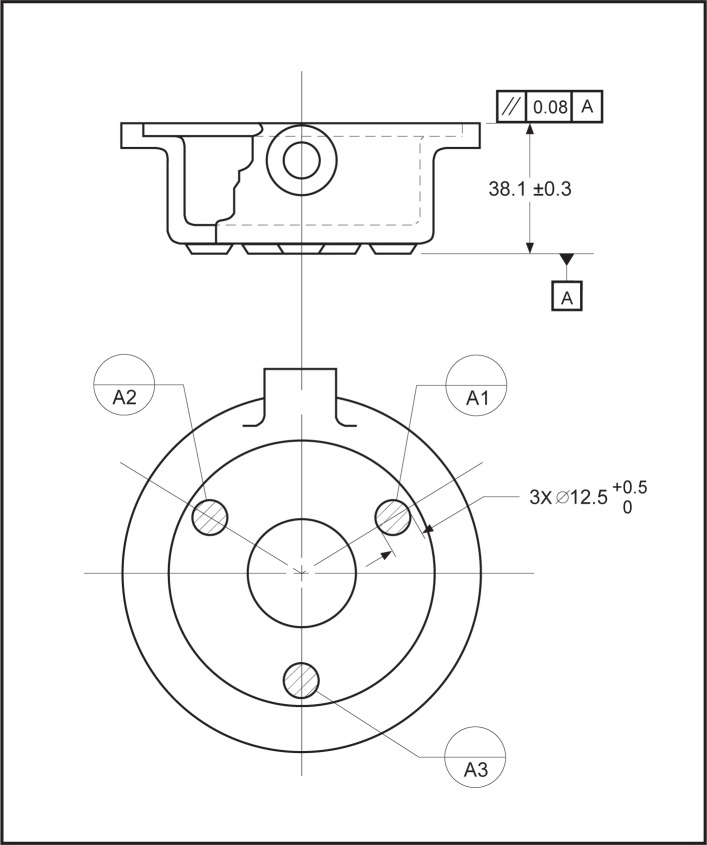
Technical drawing illustrating datum targets that are “complete features.” This figure is a reproduction of a drawing presented on page 260 in *Geo-metrics IIIm* [8].

**Fig. D.1:**
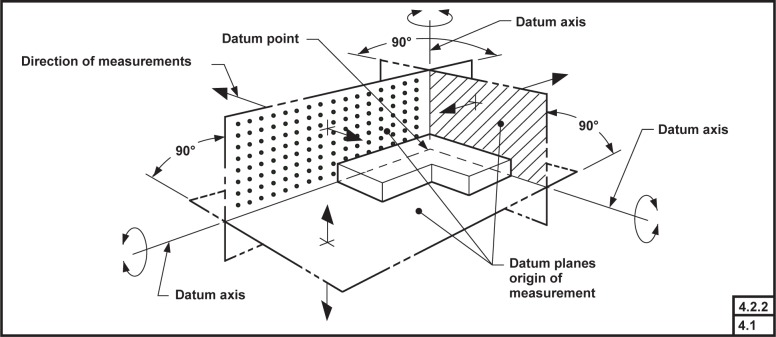
Illustration of a datum reference frame. This figure is a reproduction of FIG. 4-1 of ASME Y14.5M [4].

**Fig. E.1:**
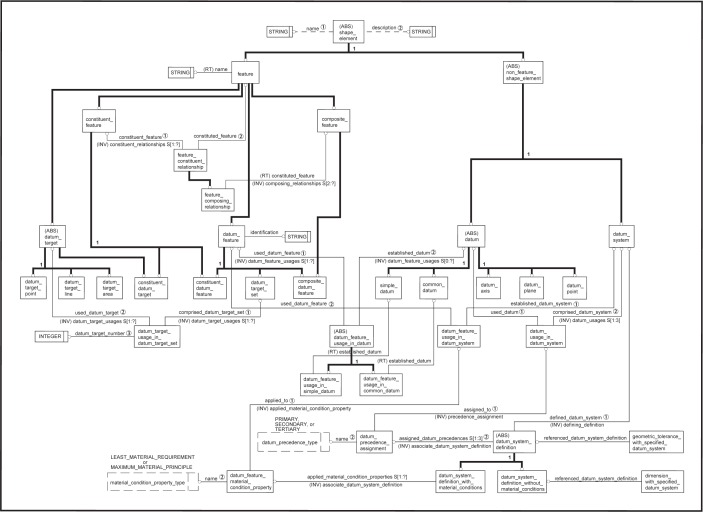
EXPRESS-G diagram that corresponds to an extended version of the DSCDM.

## 8. Appendix A. EXPRESS-G Overview

As the data models in this paper are presented in EXPRESS-G notation, this overview is provided to aid those who may be unfamiliar with EXPRESS-G.

Data models formally define data objects and relationships among data objects for a domain of interest. Some typical applications of data models include supporting the development of databases and enabling the exchange of data for a particular area of interest.

EXAMPLE—The data model presented in Fig. A.1 could be used to specify the requirements of a database for an audio compact disc (CD) collection.

Data models are specified in a data modelling language. EXPRESS is a data modelling language defined in ISO 10303-11, the *EXPRESS Language Reference Manual* [3]. EXPRESS-G is a graphical notation that supports a subset of the EXPRESS language. One of the advantages of using EXPRESS-G over EXPRESS is that the structure of a data model can be presented in a more understandable manner. A disadvantage of EXPRESS-G is that complex constraints cannot be formally specified. Figure A.2 depicts the symbols used in the EXPRESS-G notation. The meanings of these symbols are listed below.

### 8.1 Entity Data Type Symbol

Entity Data Type Symbol: An entity data type symbol [see Fig. A.2 (A)] represents a real or conceptual object of interest.

EXAMPLE—The **Person** entity in Fig. A.1 represents a person (or at least the essence of a person relevant to an audio CD database).

### 8.2 Attribute Symbol

Attribute Symbol: An attribute symbol [see Fig. A.2 (F)] represents a property of an entity. An attribute establishes a relationship between two entities or between an entity and a value.

EXAMPLE—The *title_of_CD* attribute on the **Audio_CD** entity in Fig. A.1 represents the title of an audio CD.
NOTE—The direction of the relationship is towards the bubble. However, there is also a relationship in the opposite direction. This inverse relationship is implicit unless explicitly specified with an inverse attribute. The cardinality of the implicit inverse relationship is zero or more. Explicit inverse attributes are generally specified to support EXPRESS rules or to restrict the cardinality to something other than zero or more. On EXPRESS-G diagrams the inverse attribute name is preceded by “(INV)”.
EXAMPLE—The *duration_of* inverse attribute on the **Duration** entity in Fig. A.1 indicates that a **Duration** must be specified as the *track_length* of exactly one **Music_track**. This corresponds to the assertion that a duration can only exist if it is specified as the track length of a music track.
NOTE—Attributes and optional attributes may be aggregates. Types of aggregates defined in the EXPRESS language include arrays, bags, lists, and sets. Attributes that are arrays, bags, lists, or sets are denoted on EXPRESS-G diagrams by placing the letter “A, ” “B, ” “L, ” or “S, ” respectively, after the attribute name. The size of the aggregate is indicated on the diagram within square brackets that immediately follow the letter indicating the aggregate type.
EXAMPLE—In Fig. A.1 the *track_list* attribute on the **Audio_CD** entity specifies a list of one or more (the “?” indicates that the size of the list does not have an upper bound) **Music_track**s.

### 8.3 Optional Attribute Symbol

Optional Attribute Symbol: An optional attribute symbol [see Fig. A.2 (G)] represents a property of an entity that need not be specified.

EXAMPLE—The *gender* attribute on the **Person** entity in Fig. A.1 represents the gender of a person. As it is an optional attribute, the gender of a person need not be specified.

### 8.4 Supertype/Subtype Symbol

Supertype/Subtype Symbol: A supertype/subtype symbol [see Fig. A.2 (H)] represents the relationship between an entity that is a supertype and the entity that is its subtype. The bubble is placed on the subtype entity.

EXAMPLE—In Fig. A.1 the **Instrumental** entity is a subtype of the **Music_track** entity. Conversely, the **Music_track** entity is a supertype of the **Instrumental** entity.
NOTE—A subtype inherits all the attributes of its supertype(s).
EXAMPLE—The **Instrumental** entity in Fig. A.1 inherits the *performers*, *recorded_on*, *title_of_music*, and *track_length* attributes from the **Music_track** entity.

### 8.5 Simple Data Type Symbols

Simple Data Type Symbols: Simple data type symbols [see Fig. A.2 (E)] represent basic values. The kinds of simple data types that are defined in the EXPRESS language are INTEGER, REAL, STRING, NUMBER, BOOLEAN, LOGICAL, and BINARY. The kind of simple data type determines what values the simple data type may take on.

EXAMPLE—The *band_name* attribute on the **Band** entity in Fig. A.1 is of type STRING. Therefore, it may take on a string value.

### 8.6 Select Data Type Symbol

Select Data Type Symbol: A select data type symbol [see Fig. A.2 (D)] indicates that an attribute may be one of the several types specified by the select data type.

EXAMPLE—T**he Band_or_musician_select** select data type in Fig. A.1 permits the *performers* attribute on the **Audio_CD** entity to take on values that are either of type **Band** or of type **Person**.

### 8.7 Enumerated Data Type Symbol

Enumerated Data Type Symbol: An enumerated data symbol [see Fig. A.2 (B)] indicates that values that an attribute may take on are limited to those values specified in an enumerated list.

EXAMPLE—The **Gender_type** enumerated data type in Fig. A.1 may take on a value of either MALE or FEMALE.
NOTE—The values that enumerated data types are permitted to take on are documented in EXPRESS models. These values are not normally shown in EXPRESS-G diagrams. However, for readability, the permissible values for **Gender_type** in Fig. A.1 have been annotated to the EXPRESS-G diagram.

### 8.8 Defined Data Type Symbol

Defined Data Type Symbol: A defined data type symbol [see Fig. A.2 (C)] represents a value that is based on a simple data type. However, defined data types have additional semantics associated with them and may have additional constraints placed upon them that further limit the values they may take on.

EXAMPLE—The defined type **Minute_value** in Fig. A.1 has a base type of INTEGER and has constraints placed upon it requiring that the value be less than sixty and greater than or equal to zero.
NOTE—The constraints placed on defined types are documented in EXPRESS models. These constraints are not normally shown in EXPRESS-G diagrams. However, for readability, the constraints for the defined types in Fig. A.1 have been annotated to the EXPRESS-G diagram.
NOTE—For a more complete presentation of EXPRESS and EXPRESS-G see ISO 10303-11 [3].

## 9. Appendix B. Underlying Data Model Structure

STEP integrated generic resource constructs are useful in creating product data models as they define basic concepts and the relationships between those concepts that are relevant over many domains (e.g., the relationship between a property definition and a shape aspect). The underlying structure of the DSCDM is based on four entities from the STEP integrated generic resources. These entities are **Shape_aspect**, **Shape_aspect_relationship**, **Property_definition**, and **Property_definition_relationship**. The **Shape_aspect**, **Shape_aspect_relationship**, and **Property_definition** entities are from STEP Part 41 [11]. The **Property_definition_relationship** entity is from STEP Part 45 [12]. An abbreviated EXPRESS-G diagram of these entities is shown in Fig. B.1.

The definitions of the four entities shown in Fig. B.1 are provided in Tables B.1–B.4. These definitions are taken from STEP Part 41 [11] and STEP Part 45 [12].

NOTE—The actual EXPRESS declarations of these entities have not been included in the definitions given in Tables B.1–B.4, as they are not necessary to the understanding of the concepts presented in this paper.
NOTE—The clause numbers specified within Tables B.1–B.3 are from STEP Part 41 and should not be confused with the clause numbers of this paper.
NOTE—The clause number specified within Table B.4 is from STEP Part 45 and should not be confused with a clause number of this paper.

**Table B.1:** Definition of **Shape_aspect** from STEP Part 41 [11].

**2.4.4.4 Shape_aspect**
A **Shape_aspect** is an identifiable element of the shape of a product.
EXAMPLE 36—Consider the **Product_definition_shape** of a bolt. One might distinguish, as an element of this shape, the concept of the threaded portion of its shank. This portion of the shape could be specified using a **Shape_aspect** entity so that other properties, such as surface finish, may be associated with it.
Attribute definitions:
*name*: the word or group of words by which the **Shape_aspect** is referred to.
*description*: text that relates the nature of the **Shape_aspect**.
*of_shape*: the **Product_definition_shape** of which this entity is an aspect.
EXAMPLE 37—If the identified aspect were the threaded portion of a bolt’s shank, this attribute would be the **Product_definition_shape** of the bolt.
*product_definitional*: an indication that the **Shape_aspect** is on the physical boundary of the **Product_definition_shape**. If the value of this attribute is TRUE, it is asserted that the **Shape_aspect** being identified is on such a boundary. If the value is FALSE, it shall be asserted that the **Shape_aspect** being identified is not on such a boundary. If the value is UNKNOWN, it shall be asserted that it is not known whether or not the **Shape_aspect** being identified is on such a boundary.
EXAMPLE 38—If the identified **Shape_aspect** were the threaded portion of a bolt’s shank, the value of this attribute would be TRUE. However, if it were the centre-line, the value would be FALSE.

**Table B.2:** Definition of **Shape_aspect_relationship** from STEP Part 41 [11].

**2.4.4.5 Shape_aspect_relationship**
A **Shape_aspect_relationship** is an association between two **Shape_aspect**s.
EXAMPLE 39—If one **Shape_aspect** is part of another, this entity could be used to associate the two **Shape_aspect**s.
NOTES
1—The intention is to capture distinct properties of shapes as they exist in their own right and as they participate in the relationship. The **Shape_aspect_relationship** entity is not a representation definition entity. If a **Shape_aspect_relationship** is to have an explicit representation then that is a separate concept which is described using resource constructs that are outside this schema.
2—Each **Shape_aspect** entity could have different properties.
3—No geometric relationship is established between related **Shape_aspect** entities.
EXAMPLE 40—A **Shape_aspect_relationship** might relate two **Shape_aspect**s whose representations are the equivalent surfaces of a mould and a moulded product. The shape of the mould is not spatially related to the moulded product.
NOTES
4—Relationships captured using this entity may be parent-child relationships. Specializations of this entity state this fact if it is true for a particular specialization.
5—This entity, in conjunction with the **Shape_aspect** entity, is based on the relationship template that is described in annex D.
Attribute definitions:
*name*: the word or group of words by which the **Shape_aspect_relationship** is referred to.
*description*: text that relates the nature of the **Shape_aspect_relationship**.
*relating_shape_aspect*: one of the **Shape_aspect** entities which is part of the relationship.
EXAMPLE 41—A **Shape_aspect** that is a pocket with five faces would play the role of *relating_shape_aspect* in the five **Shape_aspect_relationship** entities: one per face.
*related_shape_aspect*: the other **Shape_aspect** entity which is a part of the relationship. If one element of the relationship is dependent upon the other, this attribute shall be the dependent one.
EXAMPLE 42—In the previous example each of the five **Shape_aspect_relationship** entities would have a different **Shape_aspect** entity in the *related_shape_aspect* field. There would be one for each side and one for the bottom of the pocket.

**Table B.3:** Definition of **Property_definition** from STEP Part 41 [11].

**2.4.4.2 Property_definition**
A **Property_definition** is a property that characterizes a product. It applies to either a product, a product in the context of another product, the shape of a product, an element of the shape of a product, or an element of the shape in the context of another element of the shape of a product.
Attribute definitions:
*name*: the word or group of words by which the **Property_definition** is referred to.
*description*: text that relates the nature of the **Property_definition**.
*definition*: the **Product_definition**, **Shape_aspect**, or **Shape_aspect_relationship** whose property is identified.
NOTE—A **Property_definition** cannot exist unless it is related to either a **Product_definition** or a **Shape_definition**. This attribute establishes this relationship.

**Table B.4:** Definition of Property_definition_relationship from STEP Part 45 [12].

**4.4.2 Property_definition_relationship**
A **Property_definition_relationship** is an association between two **Property_definition**s. The meaning of the relationship for a particular context is defined in specializations of this resource construct.
NOTES
1—Relationships captured using this entity may be parent child relationships. Specializations of this entity state this fact if it is true for the particular specialization.
2—This entity, in conjunction with the **Property_definition** entity, is based on the relationship template that is described in annex D of ISO 10303-41.
Attribute definitions:
*name*: the word or group of words by which the **Property_definition_relationship** is referred to.
*description*: text that relates the nature of the **Property_definition_relationship**.
*relating_property_definition*: one of the **Property_definition**s which is a part of the relationship.
*related_property_definition*: the other **Property_definition_relationship** which is part of the relationship. If one element of the relationship is dependent upon the other, this attribute shall be the dependent one.
NOTE 3—The role of the *related_property_definition* and *relating_property_definition* attributes is defined in the part of ISO 10303 that uses or specializes this entity.

NOTE—The definition for the *related_property_definition* attribute of the **Property_definition_relationship** entity, shown in Table B.4, should read “the other **Property_definition**, ” not “the other **Property_definition_relationship**.”

## 10. Appendix C. Specializations to the DSCDM

This appendix presents possible refinements to the DSCDM. These refinements are intended to represent possible ways in which the DSCDM may be further specialized. Depending on the application, the specializations may or may not be required. Furthermore, the specializations shown here are clearly not the only way in which the DSCDM might be specialized.

NOTE—All the specializations included in this appendix have been incorporated into the data model that is presented in Appendix E. Also included in Appendix E are three data populations that are based on this extended DSCDM model.

### 10.1 Specialization of Datum

The **Datum** entity could be further refined to account for the different types of datum (i.e., datum points, datum axes, and datum planes). Figure C.1 is an EXPRESS-G diagram illustrating this specialization.

NOTE—There might be an additional constraint associated with the **Datum** entity, requiring a **Datum** to be either a **Datum_axis**, a **Datum_plane**, or a **Datum_point**.

### 10.2 Specialization of Datum_feature

Typically, when one envisions the concept of feature, one visualizes a single surface of a part. However, features may be composed of other features. Subsequently, datum features may be composite datum features.

EXAMPLE—Figures 9 and 10 show examples of composite datum features.

Furthermore, features may be constituents of other features. That is, a feature may constitute only a portion of a larger feature. Subsequently, datum features may be constituent datum features.

NOTE—On technical drawings, a “partial feature” is usually indicated with a chain line, a hatched area, or both.
EXAMPLE—The chain line in Fig. C.2 indicates that datum feature A is a constituent of the feature that is the bottom surface of the part.

In cases in which parent-child relationships need to be captured for composite and constituent datum features, the **Datum_feature** entity may be specialized, as shown in Fig. C.3.

NOTE—Entities such as **Feature** and **Feature_composing_relationship** shown in Fig. C.3 may be subtypes of more generic entities such as **Shape_element** and **Shape_element_composing_relationship**, respectively. However, for brevity, Fig. C.3 does not address these concepts.
NOTE—Figure C.3 does not show the various rules that would be associated with the entities introduced in this specialization.
EXAMPLE—A rule on the **Composite_feature** entity might correspond to the assertion that a composite feature shall not be composed of itself.

### 10.3 Specialization of Datum_target

The **Datum_target** entity could be further specialized to account for the different types of datum targets (i.e., datum target points, datum target lines, and datum target areas). Additionally, the **Datum_target** entity might be further specialized to indicate when a datum target is a constituent of another feature. Figure C.3 is an EXPRESS-G diagram illustrating this specialization.

NOTE—There might be an additional constraint associated with the **Datum_target** entity, requiring a **Datum_target** to be either a **Datum_target_point**, a **Datum_target_line**, or a **Datum_target_area**.
NOTE—Typically, datum targets are not “complete features;” typically, they are constituents of other features. However, there is no requirement that this be the case. Consequently, any specialization of the **Datum_target** entity should not require the **Datum_target** to be associated with a parent entity.
EXAMPLE—The datum targets shown in the technical drawing presented in Fig. C.4 are “complete features.”

## 11. Appendix D. Datum Reference Frame vs Datum System

This appendix discusses the difference between a datum reference frame and a datum system.

NOTE—The definition of datum reference frame is given in Sec. 3 of this paper.

Though the concept of datum reference frame is not modeled in the DSCDM (as it is outside the scope of this paper), it is important to understand how a datum reference frame differs from a datum system. While both datum reference frames and datum systems consist of datums (and some datums may be simultaneously in both datum systems and datum reference frames), the constraints placed upon the datums of a datum reference frame differs from the constraints placed upon the datums of a datum system. That is, datums of a datum system have sequential constraints (i.e., datum precedence) placed upon them. By contrast, datums of a datum reference frame have positional constraints placed upon them (e.g., the datum planes of a datum reference frame are always mutually perpendicular).

NOTE—There is no requirement that the datum planes of a datum system be mutually perpendicular.

In other words, a datum system specifies the order in which the datums are established within the datum system, and a datum reference frame specifies the positional relationship among the datums of the datum reference frame. Figure D.1 shows the inter-relationship among the datums of a datum reference frame.

NOTE—The author’s interpretation of datum reference frame is slightly different from ASME Y14.5M [4]. A sentence in clause 4.2.2.2 of ASME Y14.5M states that “any difference in the order of precedence or in the material conditions of any datum features referenced in multiple feature control frames requires different datum simulation methods and, consequently, establishes a different datum reference frame.” The author of this paper agrees with the requirement for different simulation methods. However, he believes this is because of the different datum systems. From the “data” standpoint the datums of a datum reference frame still have the same interrelational positional requirements no matter what their precedence. Therefore, only one datum reference frame is needed to specify these interrelational positional requirements.
EXAMPLE—In Fig. 10 there are two datum systems specified and only one datum reference frame. The first datum system contains datum plane A and datum axis B; and the second datum system only contains datum plane A. The datum reference frame contains datum plane A and datum axis B but it also contains the two datum planes labeled the “second and third planes of the datum reference frame.” Though not shown, the datum reference frame also contains a datum point at the intersection of the three datum planes and the two datum axes formed by the intersection of datum plane A with each of the other two datum planes.

## 12. Appendix E. Data Populations

This appendix presents three data populations that correspond to the technical drawings in Fig. 4, Fig. 10, and Fig. 12. The model used for these data populations is presented in Fig E.1 and consists of the DSCDM and the specializations of the DSCDM presented in Appendix C. This model also includes a **Shape_element** entity, which corresponds to the generic concept of shape element. Additionally, a **Non_feature_shape_element** entity is included, which corresponds to any shape element that is not actually on the surface of a part. Types of **Non_feature_shape_element**s are **Datum**s and **Datum_system**s. The data populations presented in the tables below show how the DSCDM might be used to exchange data, and also provide a validity check on the data model. These data populations are presented in the format specified in STEP Part 21, ISO 10303-21 [16].

In order to interpret the data populations presented in the tables below, it is necessary to know which values correspond to which attributes. The order in which attribute values occur within entity instances within Part 21 files (files conforming to the format specified in ISO 10303-21) follows the order in which the attributes are specified within entity declarations in the corresponding EXPRESS model. However, as the actual EXPRESS model has not been presented in this paper, Fig. E.1 has been annotated to indicate the attribute order. In cases in which an entity has multiple “forward” attributes (inverse attribute values are not explicitly specified in Part 21 files), an encircled number (e.g., ➁) has been placed after each of the attribute names to indicate the attribute’s order.

EXAMPLE—In Fig. E.1, the ➀ and ➁ symbols associated with the *used_datum* and *comprised_datum_system* attributes of the **Datum_usage_in_datum_system** entity indicate that in instances of the **Datum_usage_in_datum_system** entity, within Part 21 files, the *used_datum* attribute value would be first, followed by the *comprised_datum_system* attribute value.

EXAMPLE—Entity instance #420 in Table E.1 is an instance of the **Datum_usage_in_datum_system** entity. In this entity instance the value #340 corresponds to the *used_datum* attribute and the value #400 corresponds to the *comprised_datum_system* attribute.

NOTE—In Part 21 files, values corresponding to inherited attributes are specified prior to values corresponding to non-inherited attributes.

EXAMPLE—Entity instance #140 in Table E.2 is an instance of the **Datum_feature** entity. In this entity instance the values ‘SIDE1’, ‘right side’, and ‘A’ correspond to the *name*, *description*, and *identification* attributes, respectively. The *name* and *description* attributes are inherited from the **Shape_element** entity.

NOTE—The “$” character is used as a placeholder for optional attributes for which a value is not specified.

EXAMPLE—Entity instance #400 in Table E.1 is an instance of the **Datum_system** entity. The “$” characters in this entity instance are used to indicate that no values are specified for the optional *name* and *description* attributes that the **Datum** entity inherits from the **Shape_element** entity.

**Table E.1:** Data population that corresponds to the technical drawing presented in Fig. 4.

ISO-10303-21;
HEADER;
FILE_DESCRIPTION(('DSCDME04 data', 'DSCDME04'), '2;1');
FILE_NAME('D:\McCaleb Temp\fig04.p21', /* FILENAME */
'1998-8-23 T21:49:25', /* CREATION DATE */
('Michael McCaleb'), /* AUTHOR */
('NIST', 'Quince Orchard Rd.', 'Gaithersburg', 'MD', '20899'), /* ORGANIZATION */
'1.3.3 (22, July, 1998)', /* PROCESSOR VERSION */
'NIST EXPRESSO', /* PROCESSOR */
'Michael McCaleb'); /* AUTHORIZATION */
FILE_SCHEMA(('DSCDME04'));
ENDSEC;
DATA;
#100=FEATURE('SIDE1', 'front side');
#110=FEATURE('SIDE2', 'top side');
#120=FEATURE('SIDE3', 'left side');
#130=(CONSTITUENT_DATUM_TARGET()CONSTITUENT_FEATURE()DATUM_TARGET()DATUM_TARGET_POINT()FEATURE()SHAPE_ELEMENT('DTP1', 'front side top-right datum target'));
#140=(CONSTITUENT_DATUM_TARGET()CONSTITUENT_FEATURE()DATUM_TARGET()DATUM_TARGET_POINT()FEATURE()SHAPE_ELEMENT('DTP2', 'front side top-left datum target'));
#150=(CONSTITUENT_DATUM_TARGET()CONSTITUENT_FEATURE()DATUM_TARGET()DATUM_TARGET_POINT()FEATURE()SHAPE_ELEMENT('DTP3', 'front side bottom datum target'));
#160=(CONSTITUENT_DATUM_TARGET()CONSTITUENT_FEATURE()DATUM_TARGET()DATUM_TARGET_AREA()FEATURE()SHAPE_ELEMENT('DTA1', 'top side left datum target'));
#170=(CONSTITUENT_DATUM_TARGET()CONSTITUENT_FEATURE()DATUM_TARGET()DATUM_TARGET_AREA()FEATURE()SHAPE_ELEMENT('DTA2', 'top side right datum target'));
#180=(CONSTITUENT_DATUM_TARGET()CONSTITUENT_FEATURE()DATUM_TARGET()DATUM_TARGET_POINT()FEATURE()SHAPE_ELEMENT('DTP4', 'left side datum target'));
#190=FEATURE_CONSTITUENT_RELATIONSHIP(#130, #100);
#200=FEATURE_CONSTITUENT_RELATIONSHIP(#140, #100);
#210=FEATURE_CONSTITUENT_RELATIONSHIP(#150, #100);
#220=FEATURE_CONSTITUENT_RELATIONSHIP(#160, #110);
#230=FEATURE_CONSTITUENT_RELATIONSHIP(#170, #110);
#240=FEATURE_CONSTITUENT_RELATIONSHIP(#180, #120);
#250=DATUM_TARGET_SET('DTS1', 'three target points', 'A');
#260=DATUM_TARGET_SET('DTS2', 'two target areas', 'B');
#270=DATUM_TARGET_SET('DTS3', 'one target point', 'C');
#280=DATUM_TARGET_USAGE_IN_DATUM_TARGET_SET(#250, #130, 1);
#290=DATUM_TARGET_USAGE_IN_DATUM_TARGET_SET(#250, #140, 2);
#300=DATUM_TARGET_USAGE_IN_DATUM_TARGET_SET(#250, #150, 3);
#310=DATUM_TARGET_USAGE_IN_DATUM_TARGET_SET(#260, #160, 1);
#320=DATUM_TARGET_USAGE_IN_DATUM_TARGET_SET(#260, #170, 2);
#330=DATUM_TARGET_USAGE_IN_DATUM_TARGET_SET(#270, #180, 1);
#340=(DATUM()DATUM_PLANE()NON_FEATURE_SHAPE_ELEMENT()SHAPE_ELEMENT($, $)SIMPLE_DATUM());
#350=(DATUM()DATUM_PLANE()NON_FEATURE_SHAPE_ELEMENT()SHAPE_ELEMENT($, $)SIMPLE_DATUM());
#360=(DATUM()DATUM_PLANE()NON_FEATURE_SHAPE_ELEMENT()SHAPE_ELEMENT($, $)SIMPLE_DATUM());
#370=DATUM_FEATURE_USAGE_IN_SIMPLE_DATUM(#250, #340);
#380=DATUM_FEATURE_USAGE_IN_SIMPLE_DATUM(#260, #350);
#390=DATUM_FEATURE_USAGE_IN_SIMPLE_DATUM(#270, #360);
#400=DATUM_SYSTEM($, $);
#410=DATUM_SYSTEM($, $);
#420=DATUM_USAGE_IN_DATUM_SYSTEM(#340, #400);
#430=DATUM_USAGE_IN_DATUM_SYSTEM(#340, #410);
#440=DATUM_USAGE_IN_DATUM_SYSTEM(#350, #410);
#450=DATUM_USAGE_IN_DATUM_SYSTEM(#360, #410);
#460=DATUM_PRECEDENCE_ASSIGNMENT(#420, .PRIMARY.);
#470=DATUM_PRECEDENCE_ASSIGNMENT(#430, .PRIMARY.);
#480=DATUM_PRECEDENCE_ASSIGNMENT(#440, .SECONDARY.);
#490=DATUM_PRECEDENCE_ASSIGNMENT(#450, .TERTIARY.);
#500=DATUM_SYSTEM_DEFINITION_WITHOUT_MATERIAL_CONDITIONS(#400, (#460));
#510=DATUM_SYSTEM_DEFINITION_WITHOUT_MATERIAL_CONDITIONS(#410, (#470, #480, #490));
/* PEPENDICULARITY TOLERANCE */
#520=GEOMETRIC_TOLERANCE_WITH_SPECIFIED_DATUM_SYSTEM(#500);
/* POSITION TOLERANCE */
#530=GEOMETRIC_TOLERANCE_WITH_SPECIFIED_DATUM_SYSTEM(#510);
ENDSEC;
END-ISO-10303-21;

**Table E.2:** Data population that corresponds to the technical drawing presented in Fig. 10.

ISO-10303-21;
HEADER;
FILE_DESCRIPTION(('DSCDME04 data', 'DSCDME04'), '2;1');
FILE_NAME('D:\McCaleb Temp\fig10.p21', /* FILENAME */
'1998-8-23 T22:33:16', /* CREATION DATE */
('Michael McCaleb'), /* AUTHOR */
('NIST', 'Quince Orchard Rd.', 'Gaithersburg', 'MD', '20899'), /* ORGANIZATION */
'1.3.3 (22, July, 1998)', /* PROCESSOR VERSION */
'NIST EXPRESSO', /* PROCESSOR */
'Michael McCaleb'); /* AUTHORIZATION */
FILE_SCHEMA(('DSCDME04'));
ENDSEC;
DATA;
#100=CONSTITUENT_FEATURE('HOLE1', 'small hole top-left');
#110=CONSTITUENT_FEATURE('HOLE2', 'small hole top-right');
#120=CONSTITUENT_FEATURE('HOLE3', 'small hole bottom-left');
#130=CONSTITUENT_FEATURE('HOLE4', 'small hole bottom-right');
#140=DATUM_FEATURE('SIDE1', 'right side', 'A');
#150=COMPOSITE_DATUM_FEATURE('COMP_FEA1', 'four small holes', 'B');
#160=FEATURE_COMPOSING_RELATIONSHIP(#100, #150);
#170=FEATURE_COMPOSING_RELATIONSHIP(#110, #150);
#180=FEATURE_COMPOSING_RELATIONSHIP(#120, #150);
#190=FEATURE_COMPOSING_RELATIONSHIP(#130, #150);
#200=(DATUM()DATUM_PLANE()NON_FEATURE_SHAPE_ELEMENT()SHAPE_ELEMENT($, $)SIMPLE_DATUM());
#210=(DATUM()DATUM_AXIS()NON_FEATURE_SHAPE_ELEMENT()SHAPE_ELEMENT($, $)SIMPLE_DATUM());
#220=DATUM_FEATURE_USAGE_IN_SIMPLE_DATUM(#140, #200);
#230=DATUM_FEATURE_USAGE_IN_SIMPLE_DATUM(#150, #210);
#240=DATUM_SYSTEM($, $);
#250=DATUM_SYSTEM($, $);
#260=DATUM_USAGE_IN_DATUM_SYSTEM(#200, #240);
#270=DATUM_USAGE_IN_DATUM_SYSTEM(#200, #250);
#280=DATUM_USAGE_IN_DATUM_SYSTEM(#210, #250);
#290=DATUM_FEATURE_USAGE_IN_DATUM_SYSTEM(#250, #150);
#300=DATUM_PRECEDENCE_ASSIGNMENT(#260, .PRIMARY.);
#310=DATUM_PRECEDENCE_ASSIGNMENT(#270, .PRIMARY.);
#320=DATUM_PRECEDENCE_ASSIGNMENT(#280, .SECONDARY.);
#330=DATUM_FEATURE_MATERIAL_CONDITION_PROPERTY(#290, .MAXIUM_MATERIAL_PRINCIPLE.);
#340=DATUM_SYSTEM_DEFINITION_WITHOUT_MATERIAL_CONDITIONS(#240, (#300));
#350=DATUM_SYSTEM_DEFINITION_WITH_MATERIAL_CONDITIONS(#250, (#310, #320), (#330));
#360=GEOMETRIC_TOLERANCE_WITH_SPECIFIED_DATUM_SYSTEM(#340); /* POSITION TOLERANCE ON FOUR HOLES */
#370=GEOMETRIC_TOLERANCE_WITH_SPECIFIED_DATUM_SYSTEM(#350); /* POSITION TOLERANCE ON FITTING */
ENDSEC;
END-ISO-10303-21;

**Table E.3:** Data population that corresponds to the technical drawing presented in Fig. 12.

ISO-10303-21;
HEADER;
FILE_DESCRIPTION(('DSCDME04 data', 'DSCDME04'), '2;1');
FILE_NAME('D:\McCaleb Temp\fig12.p21', /* FILENAME */
'1998-8-22 T23:2:52', /* CREATION DATE */
('Michael McCaleb'), /* AUTHOR */
('NIST', 'Quince Orchard Rd.', 'Gaithersburg', 'MD', '20899'), /* ORGANIZATION */
'1.3.3 (22, July, 1998)', /* PROCESSOR VERSION */
'NIST EXPRESSO', /* PROCESSOR */
'Michael McCaleb'); /* AUTHORIZATION */
FILE_SCHEMA(('DSCDME04'));
ENDSEC;
DATA;
#100=DATUM_FEATURE('SHAFT1', 'larger cylindrical shaft', 'A');
#110=DATUM_FEATURE('SHAFT2', 'smaller cylindrical shaft', 'B');
#120=DATUM_FEATURE('SIDE1', 'left side', 'C');
#130=(COMMON_DATUM()DATUM()DATUM_AXIS()NON_FEATURE_SHAPE_ELEMENT()SHAPE_ELEMENT($, $));
#140=(DATUM()DATUM_PLANE()NON_FEATURE_SHAPE_ELEMENT()SHAPE_ELEMENT($, $)SIMPLE_DATUM());
#150=DATUM_FEATURE_USAGE_IN_COMMON_DATUM(#100, #130);
#160=DATUM_FEATURE_USAGE_IN_COMMON_DATUM(#110, #130);
#170=DATUM_FEATURE_USAGE_IN_SIMPLE_DATUM(#120, #140);
#180=DATUM_SYSTEM($, $);
#190=DATUM_USAGE_IN_DATUM_SYSTEM(#130, #180);
#200=DATUM_USAGE_IN_DATUM_SYSTEM(#140, #180);
#210=DATUM_FEATURE_USAGE_IN_DATUM_SYSTEM(#180, #100);
#220=DATUM_FEATURE_USAGE_IN_DATUM_SYSTEM(#180, #110);
#230=DATUM_PRECEDENCE_ASSIGNMENT(#190, .PRIMARY.);
#240=DATUM_PRECEDENCE_ASSIGNMENT(#200, .SECONDARY.);
#250=DATUM_FEATURE_MATERIAL_CONDITION_PROPERTY(#210, .MAXIMUM_MATERIAL_PRINCIPLE.);
#260=DATUM_FEATURE_MATERIAL_CONDITION_PROPERTY(#220, .MAXIMUM_MATERIAL_PRINCIPLE.);
#270=DATUM_SYSTEM_DEFINITION_WITH_MATERIAL_CONDITIONS(#180, (#230, #240), (#250, #260));
#280=GEOMETRIC_TOLERANCE_WITH_SPECIFIED_DATUM_SYSTEM(#270); /* POSITION TOLERANCE */
ENDSEC;
END-ISO-10303-21;
